# Mutations of *BRCA1*, *BRCA2*, and *PALB2* Genes in Breast Tumor Tissue: Relationship with the Effectiveness of Neoadjuvant Chemotherapy and Disease Prognosis

**DOI:** 10.3390/genes14081554

**Published:** 2023-07-28

**Authors:** Matvey M. Tsyganov, Sofia S. Sorokovikova, Elizaveta A. Lutzkaya, Marina K. Ibragimova

**Affiliations:** 1 Department of Experimental Oncology, Cancer Research Institute, Tomsk National Research Medical Center, Russian Academy of Sciences, 5, Kooperativny Street, 634050 Tomsk, Russia; sonyasoroc@yandex.ru (S.S.S.); elizavetalutzkaya@gmail.com (E.A.L.); imk1805@yandex.ru (M.K.I.); 2 Faculty of Medicine and Biology, Siberian State Medical University, 2, Moskovsky Trakt, 634050 Tomsk, Russia; 3 Biological Institute, National Research Tomsk State University, 36, Lenin Avenue, 634050 Tomsk, Russia

**Keywords:** *BRCA1*, *BRCA2*, *PALB2*, CNA, DNA copy number aberrations, mutations, efficiency, neoadjuvant chemotherapy, prognosis

## Abstract

It has been shown that the loss of function of the *BRCA1*, *BRCA2*, and *PALB2* genes due to a number of hereditary mutations or chromosomal aberrations can affect the effectiveness of chemotherapy treatment and disease prognosis in patients with various types of cancer, and in particular in breast cancer. Thus, the aim of the work was to evaluate the predictive and prognostic potential of DNA copy number aberrations and mutations in the *BRCA1*, *BRCA2*, and *PALB2* genes in breast tumors. Materials and Methods: The study included 66 patients with breast cancer. DNA copy number aberrations (CNA) were assessed by high-density CytoScanHD™ Array micro matrix analysis. Gene mutations were assessed by sequencing on the MiSeq™ Sequencing System using the Accel-Amplicon *BRCA1*, *BRCA2*, and *PALB2* Panel. Results: It has been established that the presence of a normal copy number of *PALB2* is associated with a lack of response to chemotherapy in Taxotere-containing treatment regimens (*p* = 0.05). In addition, the presence of a *PALB2* deletion is associated with 100% metastatic survival rates (log-rank test *p* = 0.04). As a result of sequencing, 25 mutations were found in the *BRCA1* gene, 42 mutations in *BRCA2*, and 27 mutations in the *PALB2* gene. The effect of mutations on the effectiveness of treatment is controversial, but an effect on the survival of patients with breast cancer has been shown. So, in the presence of pathogenic mutations in the *BRCA2* gene, 100% metastatic survival is observed (log-rank test *p* = 0.05), as well as in the elimination of *PALB2* mutations during treatment (log-rank test *p* = 0.07). Conclusion: Currently, there is little data on the effect of chromosomal aberrations and mutations in the BRCA1/2 and PALB2 genes on the effectiveness of treatment and prognosis of the disease. At the same time, the study of these genes has great potential for testing focused on a personalized approach to the treatment of patients with breast cancer.

## 1. Introduction

Breast cancer (BC) tumorigenesis is driven by various genes, among which are such classical tumor suppressors as *BRCA1* and *BRCA2* [1]. The products of these genes are phosphoproteins contributing to the process of homologous recombination repair of double DNA strand breaks, play a role in controlling the cell cycle and repression of estrogen transcriptional activity, inhibiting the proliferation of breast cells and other estrogen-dependent organs [2]. It has been noted that in the presence of heterozygous hereditary mutations in the *BRCA1* genes, the likelihood of a secondary inactivating mutation, deletion, or hypermethylation of the promoter increases, and in such cells, there is a violation of DNA repair, which leads to genomic instability, various chromosomal aberrations, and, as a result, an increased risk of developing cancer [3]. Hereditary *BRCA1* mutations increase the cumulative risk of developing breast cancer by the age of 70 to 60–70% [1]. One of the most well-known and widespread is the *BRCA1* c.5266dupC germline mutation in exon 20, which is associated with a high risk of breast cancer and ovarian cancer [4]. In addition, its relationship with the effectiveness of chemotherapy treatment has been shown, in particular, patients with the mutation are very sensitive to platinum drugs and resistant to taxanes [5]. Further studies have shown that cells with *BRCA1* and *BRCA2* dysfunction are deficient in the repair of double-stranded DNA breaks (HRD—homologous recombination deficiency) by the conservative mechanism of homologous recombination [6]. Current evidence suggests that in sporadic cancers, the processes underlying HRD may be due to more than just germline mutations (e.g., c.5266dupC, c.1961del, c.3700_3704del, c.3756_3759del, c.4035del in *BRCA1*, and c.5946del in *BRCA2*, etc.) but also by numerous other mechanisms. Tumors that are phenotypically and genetically similar to familial *BRCA1*-associated breast cancer have similar properties to those and are defined as “BRCAness” (*BRCA*-like tumors) and these common properties may be important for treatment [7].

The main reasons for the loss of activity of the *BRCA1* gene are aberrant expression, loss of heterozygosity of this gene, the presence of a deletion of the 17q21.31 locus in the tumor tissue, somatic mutations, promoter hypermethylation, etc. Relatively recently, a comparative study of the efficacy of carboplatin and docetaxel in patients with familial breast cancer and BRCA-like tumors was carried out. It was found that in BRCA-associated patients, the efficacy of carboplatin was statistically significantly higher compared to docetaxel (68% vs. 33%, respectively, *p* = 0.01). However, such an effect was not obtained in BRCAness tumors with a methylated gene promoter and underexpression [8]. Studying the predictive and prognostic values of the BRCAness tumor landscape, it was found that the status of HRD and its assessment predicted pathological complete response (PCR) in patients with triple-negative BC (*p* = 0.0012) [9]. In addition, the GeparSixto (GBG66) study found that the PCR rate in patients with triple-negative breast cancer in the general group (regardless of BRCA1/2 status) was higher in the carboplatin-treated group (RR 1.87; 95% CI 1.17–2.97; *p* = 0.009): 41.4% (60 of 145) and 56.8% (83 of 146 patients), respectively. At the same time, tumors with HRD were more likely to achieve PCR than tumors with a high level of HR (55.9% vs. 29.8%, *p* = 0.001). Moreover, patients with HRD tumors showed a higher PCR rate when carboplatin was added to the chemotherapy regimen (64.9% vs. 45.2%; *p* = 0.025) [10].

In our opinion, large chromosomal rearrangements and mutations not only in the *BRCA1* gene but also in the *BRCA2* and *PALB2* genes are of great importance in the formation of a deficiency in homologous recombination, since these genes function in a complex.

As for the *PALB2* gene, it has been found that it also plays a key role in the process of DNA repair by homologous recombination. When repairing double-strand breaks, *PALB2* bridges the BRCA complex as a “bridge” [11]. It has been shown that the loss of *PALB2* function due to mutations is an important cause of hereditary breast cancer [12,13], along with the carriage of mutations in the *BRCA1* and *BRCA2* genes [14,15]. Patients with *PALB2* mutations have more aggressive clinical and pathological features of the tumor process [16]. The effect of loss of *PALB2* function on the effectiveness of treatment was established. The objective response rate in pancreatic ductal adenocarcinoma in patients with mutations was 58% compared with 21% in the control group (*p* = 0.0022). The influence of the presence of the *PALB2* mutation on the prognosis of the disease has not yet been fully established. But, in the work of Cybulski C. et al., 116 carriers of 509_510delGA or 172_175delTTGT mutations were identified in 12,529 women diagnosed with breast cancer. Ten-year survival for *PALB2* mutations carriers and non-carriers was 48.0% (95% CI, 36.5–63.2%) and 74.7% (95% CI, 73.5–75.8%), respectively (OR 2.27, 95% CI 1.64–3.15; *p* < 0.0001) [13]. A similar result was shown for patients diagnosed with pancreatic ductal adenocarcinoma. In particular, it was found that when treated with platinum drugs, patients who carried *PALB2* mutations had a 1-year overall survival (OS) of 94% compared with 60% in the control group of patients [17]. One of the latest studies [18] showed that the use of olaparib in a patient with metastatic breast cancer with a pathogenetically significant *PALB2* mutation (exon 1, c.18G>T, p. (=)) is significantly more beneficial (median progression-free survival of 8 months), compared with patients who do not have mutations in the *PALB2* gene [19,20]. This may indicate the clinical potential of using PARP inhibitors in the presence of *PALB2* mutations.

In addition, it is interesting to study the presence of DNA copy number aberrations (CNA) of *PALB2*. Although CNA on chromosome 16 is common, no *PALB2* loss has been shown in breast cancer patients [21]. Thus, in patients with colorectal cancer, it was found that *PALB2* deletion is associated with lower relapse-free (*p* = 0.026) and overall survival (*p* = 0.028) [22]. At the same time, low expression also correlated with lower overall survival rates (at *p* < 0.001). Interestingly, in breast cancer, higher *PALB2* expression (which may be due to amplification of the 16p12.2 locus) is associated with lower overall survival (at *p* < 0.01) in stage III patients [23].

However, in the current context and the highly controversial and controversial results of many studies, it is difficult to say about the clinical significance of the presence of mutations and chromosomal aberrations of the *BRCA1*, *BRCA2*, and *PALB2* genes in a breast tumor and their impact on the effectiveness of chemotherapy and disease prognosis. In this regard, the work was aimed to evaluate CNA and mutations of the *BRCA1*, *BRCA2*, and *PALB2* genes in the breast tumor of patients and their predictive and prognostic potential.

## 2. Materials and Methods

### 2.1. Patients and Treatment

The study involved 66 patients with operable breast cancer stage IIA–IIIB (T_1–4_N_0–3_M_0_) with morphologically verified diagnosis, aged 28–68, the average age being 47.43 ± 0.78 years old (Mean ± SE) (Table 1). All of them had treatment from 2006 to 2020 at the Cancer Research Institute, Tomsk National Research Medical Center of the Russian Academy of Sciences (Tomsk, Russia). Also, the inclusion criteria for patients in the study are the appointment of neoadjuvant chemotherapy and the absence of significant comorbidities. The study was conducted according to the ethical principles suggested in the Declaration of Helsinki (fixed in 2013) and approved by the Ethical Committee of the Cancer Research Institute (Protocol No. 21 of 14 October 2022). Signed informed consent was obtained from all participants.

In accordance with the “Consensus Conference on Neoadjuvant Chemotherapy (NAC) in Carcinoma of the Breast,” on 26–28 April 2003, in Philadelphia, Pennsylvania, [24] all patients received 4–8 courses of neoadjuvant chemotherapy AC (adriamycin 50 mg/m^2^ and cyclophosphamide 600 mg/m^2^ 1 per 3 weeks), AT (adriamycin 50 mg/m^2^ and Taxotere 75 mg/m^2^) or ACT (adriamycin 50 mg/m^2^, cyclophosphamide 600 mg/m^2^, Taxotere 75 mg/m^2^), CAX (cyclophosphamide 100 mg/m^2^ intramuscularly, adriamycin 30 mg/m^2^ intravenously, and xeloda 1200 mg/m^2^ orally), CP (cyclophosphamide 1080 mg/m^2^, cisplatin 135 mg) or monotherapy Taxotere (100 mg/m^2^ 1 h infusion per day). The effect of chemotherapy was determined according to the criteria of the World Health Organization. A complete response (CR) was defined as the complete disappearance of the primary tumor and lymph node metastasis. A partial response (PR) was defined as a tumor reduction > 50% and stabilization (ST) was defined as a tumor reduction ≤ 50% or a tumor size increase of <25%. Progression (P) was described as an increase of >25% in tumor size. Conventionally, for the convenience of calculation and description, all patients were divided into two groups: patients with complete and partial regression forming a group with an objective response to neoadjuvant chemotherapy; patients with stabilization and progression forming a group with no response to treatment. After 3–5 weeks NAC all patients had a radical or subcutaneous mastectomy, radical resection, sectoral resection with axillary lymphadenectomy or other type of organ-preserving surgery; then, the patients underwent radiation and/or hormonal or targeted therapy (Herceptin for HER2+ status) according to indications. During the entire period, the patients were dynamic and monitored. The median follow-up time was 44 months (44 ± 3.75).

We analyzed biopsy tumor samples before treatment (~10 mm^3^), which were obtained under the control of an ultrasound, and after (~60–70 mm^3^) neoadjuvant treatment (after 3–5 weeks after the last course of chemotherapy). All tumor samples were placed in RNAlater solution (Thermo Scientific, Waltham, MA, USA) and stored at –80 °C (after 24 h incubation at +4 °C) for the following DNA extraction.

### 2.2. DNA Extraction

DNA was isolated from 66 paired tumor samples before and after chemotherapy with QIAamp DNA mini Kit (Qiagen, Hilden, Germany). DNA quality and concentration were assessed on a fluorometer Qubit 4.0 (Thermo Scientific, Waltham, MA, USA), from 50 to 250 ng/mcl. DNA integrity was assessed by capillary electrophoresis on a TapeStation 4150 device (Agilent Technologies, Santa Clara, CA, USA); DNA fragments were over 60 kbp.

### 2.3. Microarrays Assay

Microarray analysis on high-density microarrays from Affymetrix (USA) CytoScan^TM^ HD Array was used to evaluate the CNA performed, which contains 1 million 900 thousand non-polymorphic markers for the analysis of copy number aberrations. Sample preparation, hybridization, and scanning procedures were performed in accordance with the manufacturer’s protocol on the Affymetrix GeneChip^®^ Scanner 3000 7G system (Affymetrix, Santa Clara, CA, USA). CytoScan HD Suite has greater than 99% sensitivity and can reliably detect 25–50 kb copy number changes across the genome at high specificity (amplifications (gain) and deletions (loss) sections of chromosomes). Chromosome Analysis Suite 4.1 (Affymetrix, Santa Clara, CA, USA) was used for statistics. As a result of the analysis, amplifications (that is, an increase in the number of copies of the gene) and deletions (that is, a decrease in the number of copies of the gene) of the *BRCA1*, *BRCA2*, and *PALB2* genes were determined.

### 2.4. Next-Generation Sequencing (NGS)

*BRCA1*, *BRCA2*, and *PALB2* gene mutations in tumor and patient blood were assessed by sequencing on a MiSeq™ Sequencing System (Illumina, San Diego, CA, USA) using the Accel-Amplicon *BRCA1*, *BRCA2*, and *PALB2* Panel (Swift Biosciences, Ann Arbor, MI, USA). Libraries of tumor DNA and patient blood DNA (as a germline mutation control) were prepared using the Swift Normalase KIT (Swift Biosciences, Ann Arbor, MI, USA). Sample preparation was performed according to the kit manufacturer’s instructions and included DNA fragmentation, ligation of adapters (synthesized oligonucleotides with a known sequence) to the ends of the fragments, and amplification of the resulting libraries. Amplification was based on the bridging PCR method and the formation of clusters on a flow cell.

### 2.5. Statistics

The statistical data processing was carried out using the application package Statistica 8.0 (StatSoft Inc., Tulsa, OK, USA). Survival curves were constructed by the Kaplan–Meier method and the log-rank test for the analysis of metastatic-free survival (MFS). Frequency comparisons based on qualitative data were analyzed using Fisher’s two-sided test (http://vassarstats.net/index.html (accessed on 27 July 2023)). Data processing was performed using the GATK pipeline and genetic variants found in samples of breast tumor tissue were annotated using the ANNOVAR tool.

## 3. Results

At the first stage of the work, the CNA frequency of the *BRCA1*, *BRCA2*, and *PALB2* genes was estimated. The highest frequency of chromosome aberrations is observed in the *BRCA1* gene. The frequency of deletions and amplifications is 37.9% (25/66 patients) and 24.2% (16/66 patients), respectively. The frequency of deletions and amplifications, respectively, in the BRCA2 gene is 36.4% and 3%, and the PALB2 gene is 21.2% and 27.3%. Next, an analysis was made of the relationship between CNA of the studied genes and the main clinical and pathological characteristics of patients (age, menstrual status, histological type, tumor size, lymphogenous metastasis, histological form). It was found that none of the parameters was statistically significantly associated with the presence of chromosomal aberrations in the studied gene.

Further, it was shown that in the general group of patients with the presence of deletions (25.5%, 12/47 patients) and amplifications (31.9%, 15/47 patients) at the level of a pronounced trend (*p* = 0.07), an objective response is most often observed on treatment (complete and partial regression), compared with the group without response (stabilization and progression), where the frequency of deletions (10.5%, 2/19 patients) and amplifications (15.8%, 3/19 patients) is lower, more than twice (Table 2). It was also found that in the group of patients with stabilization and progression, *PALB2* amplification is completely absent, whereas in 8 out of 9 patients, the normal copy number of the gene is preserved, at *p* = 0.05 (Table 2).

In addition, it was found that the presence of a deletion in the *BRCA1* gene in 91% of cases determines the presence of an objective response to treatment, at *p* = 0.04.

All statistically significant associations have a borderline significance level, which can most likely be explained by a small sample for this analysis. Nevertheless, there is a tendency that, for example, the presence of a deletion (loss) of a copy of the *BRCA1* gene causes a deficiency in homologous recombination, and this is manifested in the high efficiency of platinum-containing drugs.

**Figure 1:**
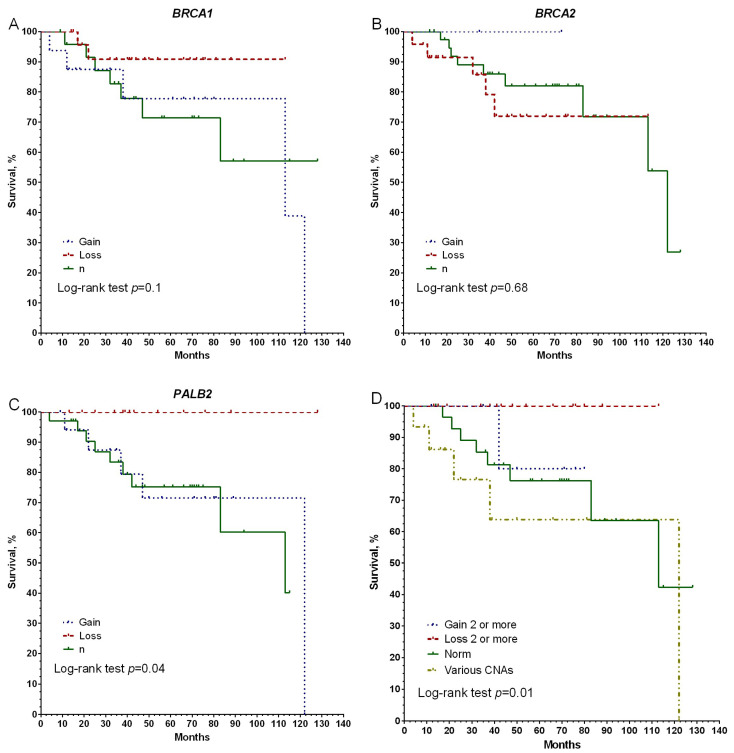
Association of chromosomal aberrations of the BRCA1, BRCA2, and PALB2 genes with metastatic-free survival. Note: Gain—amplification, Loss—deletion, n—normal copy number.

Further, using the Kaplan–Meier method, the association of MFS with the CNA of the studied genes was evaluated. In the group of 66 examined patients, distant metastases developed in 14 (21.2%) patients within 4–122 months from the moment of diagnosis. It was found that the 5-year survival rate in the group of patients with *PALB2* deletion is 100%, against 83% in the group with amplification, and 68% in the group with normal copies of this gene (log-rank test *p* = 0.04) (Figure 1C). No significant differences in survival rates were found for the *BRCA1* and *BRCA2* genes (Figure 1A,B).

It is interesting to note that if patients are divided into groups according to the presence of CNA of three genes—patients with amplifications in two or all genes, patients with deletions in two or all genes, patients with a normal copy number of genes, and a group of patients with different CNAs in three genes—then 100% survival rates are shown in the group with the presence of deletions in the studied genes compared with the norm (log-rank test *p* = 0.05) and with different CNAs (log-rank test *p* = 0.01), (Figure 1D).

**Figure 2:**
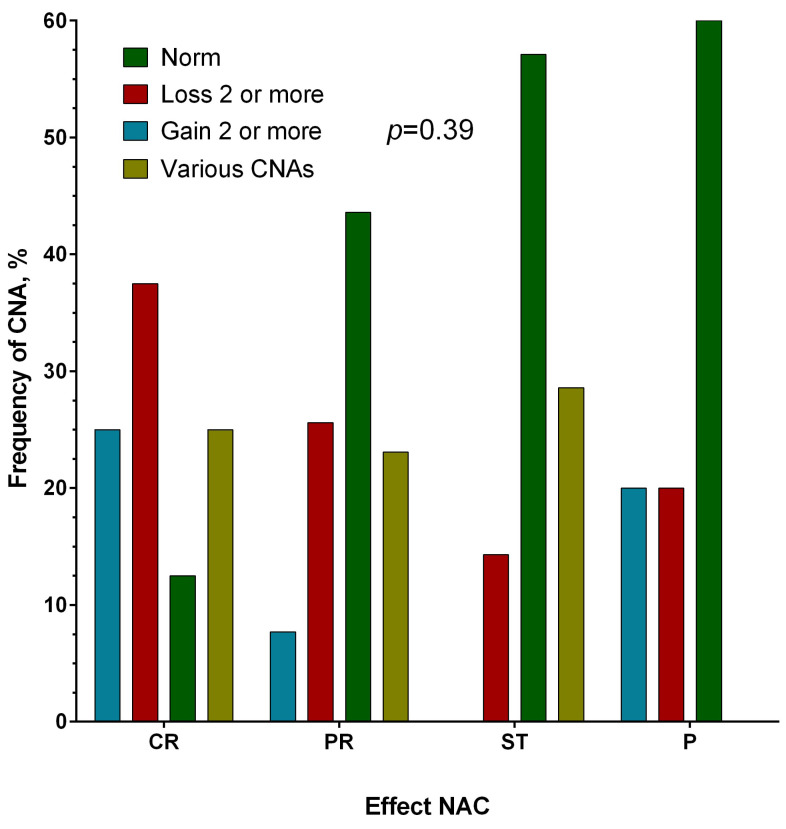
Association of the presence of cumulative chromosomal aberrations of the *BRCA1*, *BRCA2*, and *PALB2* genes with the effectiveness of neoadjuvant chemotherapy. Note: CR—complete regression; PR—partial regression; ST—stabilization; P—progression.

But at the same time, this distribution does not correlate with the effectiveness of neoadjuvant chemotherapy, *p* = 0.39 (Figure 2).

At the next stage of the work, in the formed sample of patients, using the Accel-Amplicon *BRCA1*, *BRCA2*, and *PALB2* panel, tumor samples were screened before and after chemotherapy for the presence of mutations in the studied genes.

As a result of the analysis of the *BRCA1* gene, both before and after NAC, 25 different mutations were identified (Table 3). It should be noted that synonymous SNVs do not change the amino acid sequence of a protein and, therefore, do not have a direct functional effect on its structure or function. In general, synonymous mutations are considered “neutral” in terms of functional consequences. However, studies show that not all synonymous mutations are completely neutral. Some of these may affect the rate or accuracy of transcription, mRNA splicing, or mRNA stability, which in turn may affect protein expression [25,26].

A total of 8 synonymous substitutions were identified, with a positive clinical load, as well as 12 non-synonymous substitutions, of which three mutations, c.1067A>G (rs1799950), c.2608G>A (rs753256448), and c.2609C>G (rs1060502324), have an unidentified significance.

In addition, three pathogenetically significant mutations were found: a frameshift insertion c.2612_2613insT (rs80357948), a frameshift deletion (c.4035delA (rs80357711)), and a frameshift duplication (c.5329dupC (rs397507247, rs39750724), rs431825413; rs80357906)). Moreover, the presence of c.4035delA in two patients observed the progression of the breast tumor.

**Figure 3:**
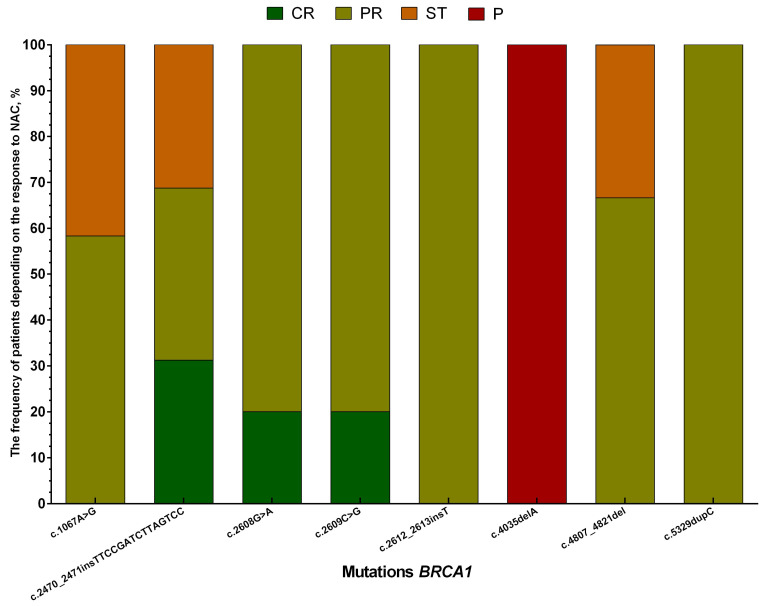
Response to neoadjuvant chemotherapy, depending on the presence of BRCA1 gene mutations in tumor biopsy material in patients with breast cancer. Note: CR—complete regression; PR—partial regression; ST—stabilization; P—progression.

In 12 patients, *BRCA1* mutations were not found both before and after NAC. For 10 patients, elimination of mutations during treatment is typical, with 80% of them having complete and partial regression of the tumor, and two progressing and stabilizing. If patients with *BRCA1* mutations that have no semantic load and clinical significance are excluded from the sample of patients, then only 8 types of mutations will remain: c.1067A>G, c.2470_2471insTTCCGATCTTAGTCC, c.2608G>A, c.2609C>G, c.2612_2613insT, c.4035delA, c.4807_4821del, and c.5329dupC. Moreover, the presence of these mutations before treatment most often determined an objective response to chemotherapy (Figure 3), in particular, this is typical for c.2608G>A, c.2609C>G, c.2612_2613insT, and c.5329dupC.

When analyzing the *BRCA2* gene, many more mutations were identified, namely 42 different mutations (Table 4). The presence of five pathogenetically significant mutations was shown, including one non-synonymous substitution and one frameshift insertion. There are 16 *BRCA2* mutations with an undetected clinical burden.

In 19 patients, mutations were not found in the biopsy material of the tumor.

**Figure 4:**
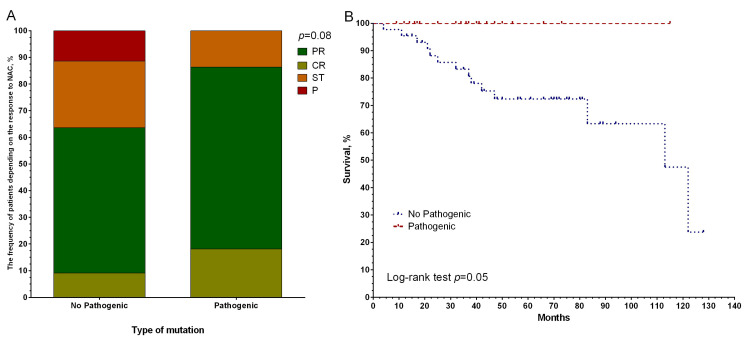
Response to neoadjuvant chemotherapy (**A**) and metastatic survival rates (**B**), depending on the presence of pathogenetically significant mutations in the BRCA2 gene in tumor biopsy material in patients with breast cancer. Note: CR—complete regression; PR—partial regression; ST—stabilization; P—progression.

Interestingly, the presence of pathogenetic *BRCA2* mutations in the tumor of breast cancer patients before treatment at the level of a pronounced trend is associated with the effectiveness of NAC (Figure 4A). In 86.4% of cases (19/22 patients), such patients have an objective response to treatment, compared with the group with stabilization and progression (63.6%, 28/44 cases), *p* = 0.08, and 100% metastatic-free survival versus 72.5% of 5-year-old MFS in the group without these mutations (log-rank test *p* = 0.05) (Figure 4B).

A total of 27 mutations were found in the *PALB2* gene spanning from exons 2 to 12, mostly concentrated in exon 8. The majority (97%, 64/66 patients) of patients had 5 intron mutations—c.3114-51T>A (rs249936); c.2586+58C>T (rs249954); c.212-58A>C (rs80291632); c.2587-38C>T (rs180177119); c.3351-53delT (rs35294437)—while the latter has an unidentified clinical significance. In addition, 8 frameshift deletions and 1 non-frameshift deletion were detected; 1 frameshift insertion and 2 non-frameshift insertions; 3 missense options; 3 non-synonymous SNVs and 3 synonymous SNVs, and one stop-gain, in a total of 26 patients (Table 5).

**Figure 5:**
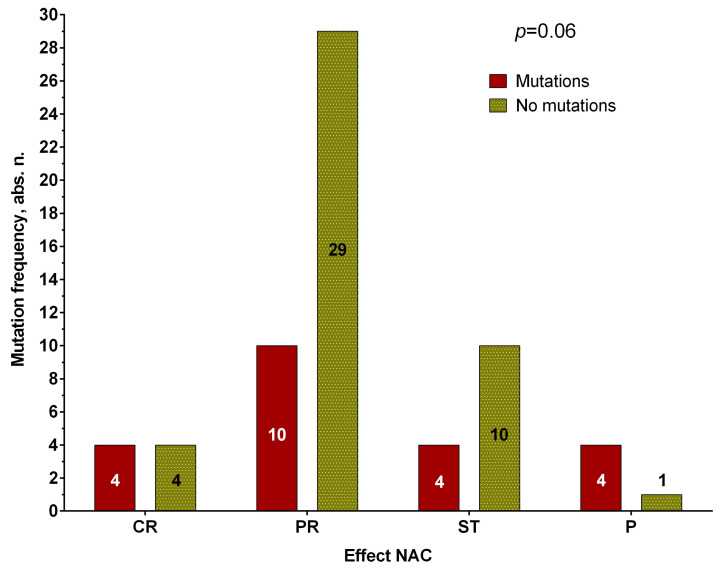
Association of the presence of *PALB2* gene mutations before treatment with the effectiveness of NAC. Note: CR—complete regression; PR—partial regression; ST—stabilization; P—progression.

The presence of non-intron mutations was detected in 33% of patients with breast cancer (22/66 cases). Moreover, the presence of these mutations in the tumor before treatment in 28.6% and 80% of cases (4/14 and 4/5) causes a lack of response to neoadjuvant chemotherapy (stabilization and progression of the tumor, respectively), the results are not statistically significant, but a relationship is shown at the level of a pronounced trend (Figure 5).

It is interesting to note that in all patients with the elimination of the frameshift deletion c.2552delA (p.Asn851fs) mutation during NAC, objective responses to treatment are observed, whereas with the appearance of this mutation, we observed the effect of chemotherapy stabilization in one patient. At the moment, the clinical significance of mutations remains unknown. Mutations c.1706_1707del (p.Lys569fs), c.1706delA (p.Lys569fs), and c.1288delC (p.Gln467fs) also occurred in patients with an objective response to treatment (Table S1).

In addition, non-synonymous SNV was most often found in the tumor biopsy material (15 out of 66 patients). Mutation c.1676A>G (p.Gln559Arg) occurred in 12 patients, of which 4 patients had complete and partial regression, and 8 patients had stabilization and progression. All identified missense mutations (c.2993G>A; c.2014G>C; c.1010T>C) were observed in patients with tumor progression during treatment. But, only c.1010T>C has pathogenic significance.

**Figure 6:**
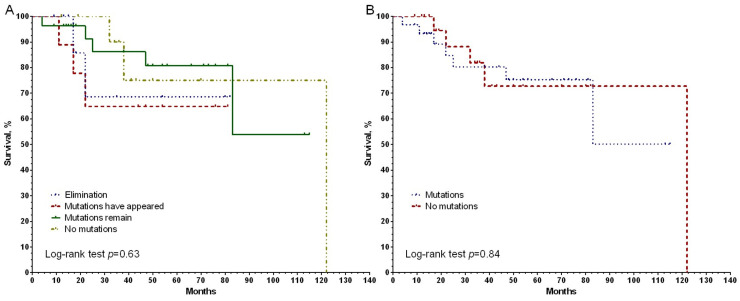

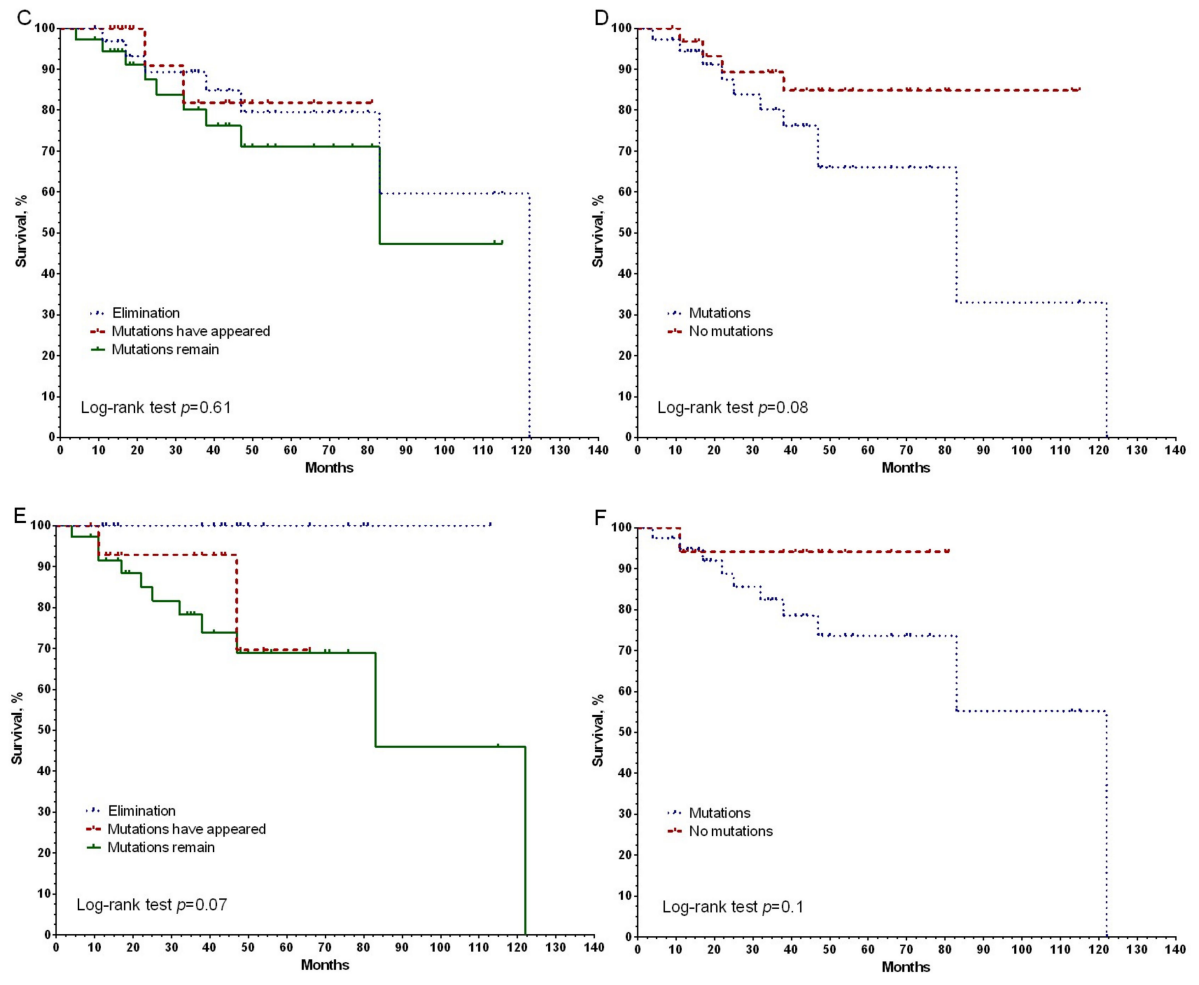
Curves of non-metastatic survival of patients with breast cancer, in accordance with the presence of *BRCA1* gene mutations during NAC (**A**) and in the surgical material (**B**), for the *BRCA2* gene (**C**,**D**) and for the *PALB2* gene (**E**,**F**).

Further, using the Kaplan–Meier method, the association of MFS with changes in the presence of mutations in the process of NAC for the three genes under study, as well as the association with the presence/absence of mutations in the surgical material of the breast tumor was assessed (Figure 6). In particular, all patients were divided into four groups: the first group consisted of patients with complete elimination of mutations during chemotherapy; the second group was of those patients who have new mutations; in the third group of patients, chemotherapy did not affect the mutation profile; the fourth group of patients had no mutations either before or after NAC. It was found that in patients with a complete absence of mutations both before and after NAC, with elimination or the appearance of new mutations during treatment, as well as in a group of patients in whom mutations were found in tumor biopsy and surgical material, the metastatic survival rates did not differ (log-rank test *p* = 0.63) (Figure 6A). When comparing groups of patients with and without *BRCA1* mutations after NAC, no statistically significant differences were found either (log-rank test *p* = 0.84) (Figure 5B). A similar result is shown for the *BRCA2* gene (Figure 6C,D). But an interesting result was demonstrated for the *PALB2* gene. So, if in the course of treatment there was an elimination of mutations of this gene in a breast tumor, then in such patients a 100% 5-year non-metastatic survival is observed, while in an unchanged state, survival does not exceed 50%. The differences are not statistically significant, but a strong trend is shown at *p* = 0.07 (Figure 6E). And consequently, the absence of mutations in the surgical material of the tumor is also at the level of a trend associated with higher rates of MFS (log-rank test *p* = 0.1).

## 4. Discussion

In our work, CNAs in the *BRCA1*, *BRCA2*, and *PALB2* genes were evaluated, as well as their clinical significance when using NAC. Such an analysis is an important promising direction for expanding the range of clinical markers when prescribing personalized therapy, since the products of the *BRCA1*, *BRCA2*, and *PALB2* genes, as mentioned above, are involved in many cell vital processes associated with maintaining genome stability. More specifically, they appear in the activation of the G2-M checkpoint, which prevents cell division in the presence of DNA damage [27], as well as in the repair of genetic information through homologous recombination.

It should be noted that according to our previous studies [28], the frequency of aberrations in the number of copies of the *BRCA1* gene in breast tumors is 53.3% (48/90 cases), of which the frequency of deletions and amplifications is 35.6% and 17.7%, respectively. which is consistent with the results obtained. It is also important that in this study, the frequency of occurrence of mutations in genes *BRCA1, BRCA2*, and *PALB2* is higher compared to the data in the literature since all classes of mutations were taken into account. The frequency of *BRCA1* and *BRCA2* deletions in breast cancer is higher than the frequency of amplifications and often represents about 10–15% of cases [29]. *BRCA1/2* amplifications are 5% [30]. It is important to note that the frequency of mutations can be dynamic, changing depending on the stage of the disease and other factors. At the same time, according to some data from the literature, mutations in the *BRCA1* and *BRCA2* genes occur in 22–28% of cases; however, these values may vary depending on the type of cancer, ethnic group, and geographic location. For example, in triple-negative breast cancer, the frequency of mutations in the *BRCA1* gene is up to 16% [31]. At the same time, the frequency of *BRCA1/2* mutations may be higher in patients with familial hereditary breast cancer—about 1–2% to 40%—depending on the population and region [32]. With regard to sporadic cases of breast cancer, in which *BRCA1* and *BRCA2* mutations occur randomly and are not associated with family history, their frequency of occurrence may be lower and range from 5% to 10% depending on the population.

Since the *BRCA1*, *BRCA2*, and *PALB2* genes are related to each other—they form physiologically significant complexes—therefore, it can be logically argued that mutations in one of the genes, leading to a change in expression, can indirectly affect the work of other proteins, and therefore, the processes in which they participate [33]. For example, there are studies where the abolition of the *BRCA1*-*PALB2* interaction led to defects in HR-based repair [34]. In our work, we observed a positive response to treatment in the group with a high frequency of gene copy number changes. That is, referring to the above facts, it can be assumed that deletions and amplifications in tumor cells could lead to their death due to a violation of the mechanisms of damage control and repair. Therefore, the presence of deletions in *BRCA1* in our study in 91% of cases was associated with a partial or complete response to the treatment with platinum-containing drugs.

In the *BRCA1* gene, we detected a pathogenic frameshift deletion c.4035delA in two people with progression. This mutation is described in the literature as a founder mutation [35]. Overall, a number of meta-analyses have shown that the addition of platinum to neoadjuvant chemotherapy regimens increases PCR rates in a group with a *BRCA* mutation compared with wild-type patients, but this phenomenon is at the level of a trend and does not reach statistical significance. Among mutant *BRCA1* patients, 58.4% (93/159 cases) were found to have achieved PCR, while only 50.7% of wild-type cases (410/808 patients) showed PCR (OR 1.459 CI 95% [0.953–2.34], *p* = 0.082) [36]. Another meta-analysis found that the cumulative pathological complete response rates were 43.4% (59/136) and 33.9% (77/227) for the platinum-treated and control group, respectively. The addition of platinum to the neoadjuvant regimen also did not lead to a significant improvement in the PCR rate (odds ratio [OR]: 1.340, 95% confidence interval [CI] = 0.677–2.653, *p* = 0.40) [37].

Interestingly, among the *BRCA2* gene mutations, the c.8208_8209insAG (rs483353122) mutation was found, which was not previously described in the 1000G, ExAC, SIFT, PolyPhen2, Mutation Taster databases. In dbSNP, ENIGMA rs483353122 of the *BRCA2* gene is classified as a highly pathogenic frameshift mutation. Moreover, this mutation was discovered by our colleagues [38], and the question of its clinical significance remains open. A pathogenetically significant BRCA2 mutation c.8673_8674delAA was identified only in prostate cancer patients without an aggressive course of this disease [39]. There is little information about the effect of c.9090delA mutations, it is reported that it was found in colorectal cancer [40], and it can also determine sensitivity to PARP inhibitors [41].

Currently, there is little risk of familial breast cancer development in patients with mutations in the *PALB2* gene. It has been established that mutations altering the gene protein are scattered throughout the coding region [14,42]; however, four *PALB2* mutations are most common: *PALB2* c.509_510delGA (p.Arg170fs*14) [43], *PALB2* c.2323C>T (p.Gln775*) [44], *PALB2* c.1592delT (p.531 fs*30) [45], and *PALB2* c.3113G>A, [46,47].

It has been shown that mutations in *PALB2*, which binds *BRCA2*, affect the double-strand break repair mechanism [48], which provides a basis for the targeted use of chemotherapeutic agents targeting this mechanism. Some authors have shown the effectiveness of mitomycin C and/or cisplatin in the presence of mutations in the gene under study [49]. It has been shown that the presence of somatic mutation T413S in small-cell lung cancer disrupts the association of the gene with chromatin and reduces resistance to camptothecin [50]. There is also evidence that high expression of *PALB2* in non-small cell lung cancer is associated with overall survival (at *p* = 0.0266) [51]. In addition, high expression of *PALB2* mRNA was a predictor of response to cisplatin/docetaxel chemotherapy. In particular, in these patients, the objective response rate was 77% compared to the low expression group, where the response rate was only 23% (*p* = 0.0448). Whereas in acute myeloid leukemia, high expression of PALB2, on the contrary, was associated with worse OS parameters [52].

In our study, it was found that the frequency of occurrence of mutations in the *PALB2* gene is 8 frameshift deletions and 1 non-frameshift deletion; 1 frameshift insertion and 2 non-frameshift insertion; 3 missense options; 3 non-synonymous SNVs and 3 synonymous SNVs, and one stop-gain. Among the frameshift deletions, two mutations (c.1706_1707del and c.1686delG) are of pathogenic significance. Many authors suggest that these mutations may contribute to the risk of familial breast cancer [14,53]. For other mutations of this class, clinical significance remains unidentified. Interestingly, three missense variants found in our sample, c.2993G>A (p.Gly998Glu); c.2014G>C (p.Glu672Gln) and c.1010T>C (p.Leu337Ser) were described earlier [54,55]. Moreover, some authors suggest that c.1010T>C (p.Leu337Ser) and c.2993G>A (p.Gly998Gln) can affect the function of the PALB2 protein [56]. 

The most common variant, c.1676A>G (Gln559Arg; rs152451), which is predicted by CADD, Condel, PolyPhen2, and SIFT to be benign, was significantly more common in cases (19.9% carried at least one non-wild-type allele) than controls (16.8% non-wild-type) [57]. It was found that the c.94C>G (p.Leu32Val) mutation was observed in several patients with familial breast cancer [47,58]. 

As for the association of mutations with disease prognosis in patients with breast cancer, it has been shown that the presence of mutations in the gene under study is not associated with disease-free survival [59]. For pancreatic ductal adenocarcinoma, platinum-treated patients who had *PALB2* mutations had a higher overall survival (mean: 20.1 months) compared to non-mutated controls (mean: 15.5 months), (*p* = 0.002). At the same time, in patients who did not receive platinum-containing drugs, there were no significant differences in survival between groups (at *p* = 0.12), [17]. In our case, the presence of mutations in the PALB2 gene in patients treated according to the cyclophosphamide/cisplatin NAC regimen did not affect the effectiveness of treatment (*p* = 0.49) and the prognosis of the disease (log-rank test *p* = 1.00) (data not shown). 

In a study by K. Pylkäs on the Finnish population, it was shown that patients with breast cancer and ovarian cancer did not reveal large genomic rearrangements, in particular deletions [29], which is confirmed by another study [44]. In our work, it was found that in almost 50% of cases of patients with breast cancer, chromosomal aberrations of *PALB2* are observed. Thus, even though the frequency of mutations in the *PALB2* gene is rare (from 0.1% to 2.7%), the risk of developing breast cancer, at least for some *PALB2* mutations, remains high.

To this day, there is little data on the impact of large chromosomal rearrangements and some previously undiscovered mutations in the *BRCA1/2* and *PALB2* genes on the effectiveness of treatment and prognosis of the disease. At the same time, the study of these genes has great potential for testing focused on diagnosis, prevention, and a personalized approach to the treatment of cancer patients.

## Figures and Tables

**Table 1:** Clinical and pathological parameters of breast cancer patients.

Clinical and Pathological Parameter		Number of Patients, abs. (%)
Age	≤45	31 (47.0)
	>45	35 (53.0)
Menstrual status	Perimenopause	35 (53.0)
	Postmenopause	31 (47.0)
Histological type	Invasive ductal cancer	45 (68.2)
	Invasive lobular carcinoma	21 (31.8)
Size	T_1_	7 (10.6)
	T_2_	53 (80.4)
	T_3_	3 (4.5)
	T_4_	3 (4.5)
Lymphogenous metastasis	N_0_	25 (37.9)
	N_1_	31 (47.0)
	N_2_	7 (10.6)
	N_3_	3 (4.5)
Histological form	Unicentric	31 (47.0)
	Multicentric	35 (53.0)
Molecular subtype	Luminal B HER2-negative	51 (77.3)
	HER2+	5 (7.6)
	Triple-negative	10 (15.2)
NAC scheme	CAX	11 (16.7)
	AC	12 (18.2)
	Taxotere	21 (31.8)
	AT/ACT	8 (12.1)
	CP	14 (21.2)
NAC effect	Complete regression	8 (12.1)
	Partial regression	39 (59.1)
	Stabilization	14 (21.2)
	Progression	5 (7.6)

Note: TNM classification: T—tumor; N—nodulus; NAC—neoadjuvant chemotherapy; CAX—cyclophosphamide/adriamycin/xeloda; AC—adriamycin/cyclophosphamide; AT—adriamycin/Taxoter; ACT—adriamycin/cyclophosphamide/Taxoter; CP—cyclophosphamide/cisplatin.

**Table 2:** Association of the presence of chromosomal aberrations in the BRCA1, BRCA2, and PALB2 genes with the effectiveness of neoadjuvant chemotherapy.

Gene/CNA	*BRCA1*		*BRCA2*		*PALB2*	
	Effect of NAC/Scheme of NAC					
	All Patients (abs. Number, %)					
	CR + PR	ST + P	CR + PR	ST + P	CR + PR	ST + P
Gain	11 (23.4)	5 (26.3)	1 (2.1)	1 (5.3)	15 (31.9)	3 (15.8)
Loss	21 (44.7)	4 (21.1)	15 (31.9)	9 (47.4)	12 (25.5)	2 (10.5)
n	15 (31.9)	10 (52.6)	31 (66)	9 (47.4)	20 (42.6)	14 (73.7)
*p*-level	0.16		0.19		0.07	
	CAX (abs. number, %)					
Gain	3 (42.9)	3 (75.0)	0 (0)	1 (25.0)	2 (28.6)	1 (25.0)
Loss	2 (28.6)	0 (0)	4 (57.1)	3 (75.0)	2 (28.6)	0 (0)
n	2 (28.6)	1 (25.0)	3 (42.9)	0 (0)	3 (42.9)	3 (75.0)
*p*-level	0.44		0.16		0.44	
	AC (abs. number, %)					
Gain	1 (11.1)	1 (33.3)	1 (11.1)	0 (0)	3 (33.3)	2 (66.7)
Loss	2 (22.2)	0 (0)	3 (33.3)	1 (33.3)	3 (33.3)	0 (0)
n	6 (66.7)	2 (66.7)	5 (55.6)	2 (66.7)	3 (33.3)	1 (33.3)
*p*-level	0.51		0.82		0.44	
	Taxotere/AT/ACT (abs. number, %)					
Gain	6 (30.0)	1 (11.1)	0 (0)	0 (0)	8 (40.0)	0 (0)
Loss	7 (35.0)	3 (33.3)	4 (20.0)	4 (44.4)	3 (15.0)	1 (11.1)
n	7 (35.0)	5 (55.6)	16 (80.0)	5 (55.6)	9 (45.0)	8 (88.9)
*p*-level	0.45		0.39		0.05	
	CP (abs. number, %)					
Gain	1 (9.1)	0 (0)	0 (0)	0 (0)	2 (18.2)	0 (0)
Loss	10 (90.9)	1 (33.3)	4 (36.4)	1 (33.3)	4 (36.4)	1 (33.3)
n	0 (0)	2 (66.7)	7 (63.6)	2 (66.7)	5 (45.5)	2 (66.7)
*p*-level	0.04		0.99		0.36	

Note: CR—complete regression; PR—partial regression; ST—stabilization; P—progression. Abs. number—absolute number. CR + PR—a group of patients with response to neoadjuvant chemotherapy; ST + P—a group of patients without response to neoadjuvant chemotherapy. Gain—amplification, Loss—deletion, n—normal copy number. Statistically significant differences are highlighted in bold.

**Table 3:** Mutations of the *BRCA1* gene in breast tumors.

№	Mutation	rs	Class	Type
1	c.1067A>G (p.Gln356Arg)	rs1799950	Non-synonymous SNV	VUS
2	c.1114A>C (p.Asn372His)	rs144848	Non-synonymous SNV	Little Clinical Significance
3	c.2077G>A (p.Asp693Asn)	rs4986850	Non-synonymous SNV	Benign
4	c.2082C>T (p.Ser694=)	rs1799949	Synonymous SNV	Benign
5	c.2311T>C (p.Leu771=)	rs16940	Synonymous SNV	Benign
6	c.2470_2471insTTCCGATCTTAGTCC (p.Pro 824delinsLPILVP)	-	Non-frameshift insertion	VUS
7	c.2608G>A (p.Ala870Thr)	rs753256448	Non-synonymous SNV	VUS
8	c.2609C>G (p.Ala870Gly)	rs1060502324	Non-synonymous SNV	VUS
9	c.2612_2613insT (p.Phe872fs)	rs80357948	Frameshift insertion	Pathogenic
10	c.2612C>T (p.Pro871Leu)	rs799917	Non-synonymous SNV	Benign
11	c.3113A>G (p.Glu1038Gly)	rs16941	Non-synonymous SNV	Benign
12	c.3396A>G (p.Lys1132=)	rs1801406	Synonymous SNV	Little Clinical Significance
13	c.3548A>G (p.Lys1183Arg)	rs16942	Non-synonymous SNV	Benign
14	c.4035delA (p.Glu1345fs)	rs80357711	Frameshift deletion	Pathogenic
15	c.4308T>C (p.Ser1436=)	rs1060915	Synonymous SNV	Benign
16	c.4563A>G (p.Leu1521=)	rs206075	Synonymous SNV	Benign
17	c.4807_4821del (p.Pro1603_Val1607del)	rs80359888	Non-frameshift deletion	VUS
18	c.4837A>G (p.Ser1613Gly)	rs1799966	Non-synonymous SNV	Benign
19	c.4900A>G (p.Ser1634Gly)	rs1799966	Non-synonymous SNV	Benign
20	c.4946T>C (p.Met1649Thr)	rs4986854	Non-synonymous SNV	Benign
21	c.5019G>A (p.Met1673Ile)	rs1799967	Non-synonymous SNV	Benign
22	c.5329dupC (p.Gln1777fs)	rs397507247, rs397507246, rs431825413; rs80357906	Frameshift duplication	Pathogenic
23	c.6513G>C (p.Val2171=)	rs206076	Synonymous SNV	Benign
24	c.7242A>G (p.Ser2414=)	rs1799955	Synonymous SNV	Little Clinical Significance
25	c.999T>C (p.Ser333=)	-	Synonymous SNV	Benign

Note: VUS—variants of uncertain significance.

**Table 4:** Mutations of the *BRCA2* gene in breast tumors.

№	Mutation	rs	Class	Type
1	c.10076A>G (p.Glu3359Gly)	rs80358389	Non-synonymous SNV	VUS
2	c.10234A>G (p.Ile3412Val)	rs1801426	Non-synonymous SNV	Benign
3	c.1114A>C (p.Asn372His)	rs144848	Non-synonymous SNV	Little Clinical Significance
4	c.125A>G (p.Tyr42Cys)	rs4987046	Non-synonymous SNV	Benign
5	c.1295A>T (p.Glu432Val)	-	Non-synonymous SNV	VUS
6	c.1305A>G (p.Arg435=)	-	Synonymous SNV	VUS
7	c.1309_1310insAAATC	-	Frameshift insertion	VUS
8	c.1365A>G (p.Ser455=)	rs1801439	Synonymous SNV	Benign
9	c.1514T>C (p.Ile505Thr)	rs28897708	Non-synonymous SNV	Benign
10	c.2229T>C (p.His743=)	rs1801499	Synonymous	Benign
11	c.-26G>A	rs1799943	5 prime UTR	VUS
12	c.2971A>G (p.Asn991Asp)	rs1799944	Non-synonymous SNV	Benign
13	c.3396A>G (p.Lys1132=)	rs1801406	Synonymous SNV	Little Clinical Significance
14	c.3807T>C (p.Val1269=)	rs543304	Synonymous SNV	Likely Benign
15	c.3823_3824insAGCAGTTCC (p.Ile1275delinsKQFL)	-	Frameshift insertion	VUS
16	c.3824_3825del p.(Ile1275ArgfsTer4)	-	Frameshift deletion	Pathogenic
17	c.3824delT (p.Ile 1275fs)	-	Frameshift deletion	VUS
18	c.4068G>A (p.Leu1356=)	rs28897724	Synonymous SNV	Benign
19	c.4258G>T (p.Asp1420Tyr)	rs28897727	Non-synonymous SNV	Benign
20	c.4288_4289insGGAACTGAGT (p.Thr1430_1431Ala_delinsRNX)	-	Stop-gain	VUS
21	c.4563A>G (p.Leu1521=)	rs206075	Synonymous SNV	Benign
22	c.5455C>T (p.Pro1819Ser)	rs80358768	Non-synonymous SNV	Benign
23	c.5744C>T (p.Thr1915Met)	rs4987117	Non-synonymous SNV	Benign
24	c.590C>G (p.Ser197Cys)	rs876659940	Non-synonymous SNV	VUS
25	c.6513G>C (p.Val2171=)	rs206076	Synonymous SNV	Benign
26	c.6577G>C (p.Glu2193Gln)	-	Non-synonymous SNV	VUS
27	c.7108_7109insCAT (p.K2370delinsTX)	-	Stop-gain	VUS
28	c.7110_7111insATATGTGGG (p.Lys2370delinsKICG)	-	Non-frameshift insertion	VUS
29	c.7242A>G (p.Ser2414=)	rs1799955	Synonymous SNV	Little Clinical Significance
30	c.7397T>C (p.Val2466Ala)	rs169547	Non-synonymous SNV	Benign
31	c.7544C>T (p.Thr2515Ile)	rs28897744	Non-synonymous SNV	Benign
32	c.8208_8209insAG (p.Leu2737fs)	rs483353122	Frameshift insertion	Pathogenic
33	c.865A>C (p.Asn289His)	rs766173	Non-synonymous SNV	Benign
34	c.8673_8674delAA (p.Thr2891fs)	rs80359724	Frameshift deletion	Pathogenic
35	c.8878C>G (p.Gln2960Glu)	-	Non-synonymous SNV	VUS
36	c.8878C>T p.(Gln2960Ter)	-	Non-synonymous SNV	Pathogenic
37	c.8881_8884del (p.Gly2961fs)	-	Frameshift deletion	VUS
38	c.8885T>A	-	Stop-gain	VUS
39	c.9038C>T (p.Thr3013Ile)	rs28897755	Non-synonymous SNV	Benign
40	c.9090delA (p.T3030fs)	rs397507420	Frameshift deletion	Pathogenic
41	c.9090dupA (p.Thr3030fs)	-	Frameshift duplication	VUS
42	c.9976A>T (p.Lys3326*)	rs11571833	Stop-gain	Benign

Note: VUS—variants of uncertain significance.

**Table 5:** Mutations of the *PALB2* gene in breast tumors.

№	Mutation	rs	Class	Type
1	c.1010T>C (p.Leu337Ser)	rs45494092	Non-synonymous SNV	Benign
2	c.1288delC (p.Gln467fs)	-	Frameshift deletion	VUS
3	c.1675_1676insGAGTGAAAGGTAA ATCAAGATGTGTGCTCTTCCGACTCC (p.Q559delinsRVKGKSRCVLFRLQ)	-	Non-frameshift insertion	VUS
4	c.1676A>G (p.Gln559Arg)	rs152451	Non-synonymous SNV	Benign
5	c.1686delG (p.Gly562fs)	-	Frameshift deletion	Pathogenic
6	c.1693delA (p.Ser565fs)	-	Frameshift deletion	VUS
7	c.1706_1707del (p.Lys569fs)	rs1060502759	Frameshift deletion	Pathogenic
8	c.1706delA (p.Lys569fs)	-	Frameshift deletion	VUS
9	c.2014G>C (p.Glu672Gln)	rs45532440	Non-synonymous SNV	Benign
10	c.212-58A>C	rs80291632	Intronic	Benign
11	c.2552delA (p.Asn851fs)	-	Frameshift deletion	VUS
12	c.255dupA (p.Thr86fs)	-	Frameshift duplication	VUS
13	c.256delA (p.Thr86fs)	-	Frameshift deletion	VUS
14	c.2586+58C>T	rs249954	Intronic	Benign
15	c.2587-38C>T	rs180177119	Intronic	Likely benign
16	c.2993G>A (p.Gly998Glu)	rs45551636	Non-synonymous SNV	Benign
17	c.3114-51T>A	rs249936	Intronic	Benign
18	c.3300T>G (p.Thr1100=)	rs45516100	Synonymous SNV	Benign
19	c.3351-53delT	rs35294437	Intronic	VUS
20	c.3465dupT (p. Asp1156 Gln1157delinsX)	-	Stop-gain	VUS
21	c.747T>A (p.Pro249=)	-	Synonymous SNV	VUS
22	c.833T>A (p.Leu278Gln)	rs200843485	Non-synonymous SNV	VUS
23	c.834A>T (p.Leu278=)	rs199919863	Synonymous SNV	Likely benign
24	c.92_94del (p.31_32del)	-	Non-frameshift deletion	VUS
25	c.921delA (p. Lys307fs)	rs202151522	Frameshift deletion	VUS
26	c.94C>G (p.Leu32Val)	rs151316635	Non-synonymous SNV	Conflicting interpretations of pathogenicity
27	c.95_96insCGGAAG (p.Leu32 delinsLGR)	-	Non-frameshift insertion	VUS

Note: VUS—variants of uncertain significance.

## Data Availability

Certificate of state registration of the database No. 2022621615 dated 6 July 2022 “Database of the results of sequencing of the *BRCA1* and *BRCA2* genes in patients with luminal B breast cancer treated with platinum preparations” Tsyganov M.M., Ibragimova M.K., Garbukov E.Yu., Usynin E.A., Litvyakov N.V. Certificate of state registration of the database No. 2022620759 dated 6 April 2022 “Database of the genetic landscape of breast cancer patients with a triple negative phenotype” Ibragimova M.K., Tsyganov M.M., Garbukov E.Yu., Zdereva E.A., Usynin E.A., Litvyakov N.V. Certificate of state registration of the database No. 2022621573 dated 4 July 2022 “Database of the results of sequencing the *PALB2* gene in patients with luminal B breast cancer” Tsyganov M.M., Ibragimova M.K., Garbukov E.Yu., Usynin E.A., Litvyakov N.V.

## Supplementary Materials

The following supporting information can be downloaded at: https://www.mdpi.com/article/10.3390/genes14081554/s1. Table S1: Mutations of the *BRCA1, BRCA2* and *PALB2* genes in breast tumors of patients before and after neoadjuvant chemotherapy.
