# Supplementary material for: Mutations of BRCA1, BRCA2, and PALB2 Genes in Breast Tumor Tissue: Relationship with the Effectiveness of Neoadjuvant Chemotherapy and Disease Prognosis
Source: Genes (Basel). 2023 Jul 28;14(8):1554. doi: 10.3390/genes14081554 (PMC10454606; doi:10.3390/genes14081554)
Supplement: Supplementary file 1 [file genes-14-01554-s001.zip › genes-2485726-supplementary.pdf]

Supplementary Material

Table S1 - Mutations of the BRCA1, BRCA2 and PALB2 genes in breast tumors of patients before and after neoadjuvant chemotherapy.

Patient	NAC scheme	Efficiency of NAC		Before NAC				After NAC			
		%	Effect	Mutation	rs	Class	Type	Mutation	rs	Class	Type
B1	AC	64	PR	***BRCA1***							
				c.1067A>G (p.Gln356Arg)	rs1799950	Non-synonymous SNV	VUS	c.1067A>G (p.Gln356Arg)	rs1799950	Non-synonymous SNV	VUS
				BRCA2							
				c.3396A>G (p.Lys1132=)	rs1801406	Synonymous SNV	Little Clinical Significance	Elimination			
				c.4563A>G (p.Leu1521=)	rs206075	Synonymous SNV	Benign				
				c.6513G>C (p.Val2171=)	rs206076	Synonymous SNV	Benign				
				c.1114A>C (p.Asn372His)	rs144848	Non-synonymous SNV	Little Clinical Significance				
				c.7397T>C (p.Val2466Ala)	rs169547	Non-synonymous SNV	Benign				
				c.9038C>T (p.Thr3013Ile)	rs28897755	Non-synonymous SNV	Benign				
				PALB2							
				c.3114-51T>A	rs249936	Intronic	Benign	c.3114-51T>A	rs249936	Intronic	Benign
				c.2586+58C>T	rs249954	Intronic	Benign	c.2586+58C>T	rs249954	Intronic	Benign
B2	AC	75	PR	***BRCA1***							
				c.2612C>T (p.Pro871Leu)	rs799917	Non-synonymous SNV	Benign	c.2612C>T (p.Pro871Leu)	rs799917	Non-synonymous SNV	Benign
				BRCA2							
				c.4563A>G (p.Leu1521=)	rs206075	Synonymous SNV	Benign	c.4563A>G (p.Leu1521=)	rs206075	Synonymous SNV	Benign
				c.6513G>C (p.Val2171=)	rs206076	Synonymous SNV	Benign	c.6513G>C (p.Val2171=)	rs206076	Synonymous SNV	Benign
				c.7397T>C (p.Val2466Ala)	rs169547	Non-synonymous SNV	Benign	c.7397T>C (p.Val2466Ala)	rs169547	Non-synonymous SNV	Benign
				c.4563A>G (p.Leu1521=)	rs206075	Synonymous SNV	Benign	c.4563A>G (p.Leu1521=)	rs206075	Synonymous SNV	Benign
				PALB2							
				c.3114-51T>A	rs249936	Intronic	Benign	c.3114-51T>A	rs249936	Intronic	Benign
D1	AC	100	CR	***BRCA1***							
				c.4308T>C (p.Ser1436=)	rs1060915	Synonymous SNV	Benign	Elimination			
				c.2311T>C (p.Leu771=)	rs16940	Synonymous SNV	Benign				
				c.2082C>T (p.Ser694=)	rs1799949	Synonymous SNV	Benign				
				c.4900A>G (p.Ser1634Gly)	rs1799966	Non-synonymous SNV	Benign				
				c.3548A>G (p.Lys1183Arg)	rs16942	Non-synonymous SNV	Benign				
				c.3113A>G (p.Glu1038Gly)	rs16941	Non-synonymous SNV	Benign				
				c.2612C>T (p.Pro871Leu)	rs799917	Non-synonymous SNV	Benign				
				BRCA2							
				c.3396A>G (p.Lys1132=)	rs1801406	Synonymous SNV	Little Clinical Signific	Elimination			

							ance				
				c.4563A>G (p.Leu1521=)	rs206075	Synonymous SNV	Benign				
				c.6513G>C (p.Val2171=)	rs206076	Synonymous SNV	Benign				
				c.4258G>T (p.Asp1420Tyr)	rs28897727	Non-synonymous SNV	Benign				
				c.7397T>C (p.Val2466Ala)	rs169547	Non-synonymous SNV	Benign				
				PALB2							
				c.3114-51T>A	rs249936	Intronic	Benign	Elimination			
E1	AC	65	PR	***BRCA1***							
				No mutations				No mutations			
				BRCA2							
				c.3396A>G (p.Lys1132=)	rs1801406	Synonymous SNV	Little Clinical Significance	Elimination			
				c.4563A>G (p.Leu1521=)	rs206075	Synonymous SNV	Benign				
				c.6513G>C (p.Val2171=)	rs206076	Synonymous SNV	Benign				
				c.7242A>G (p.Ser2414=)	rs1799955	Synonymous SNV	Little Clinical Significance				
				c.7397T>C (p.Val2466Ala)	rs169547	Non-synonymous SNV	Benign				
				PALB2							
				c.3114-51T>A	rs249936	Intronic	Benign	c.3114-51T>A	rs249936	Intronic	Benign
E2	AC	-66	P	***BRCA1***							
				c.4308T>C (p.Ser1436=)	rs1060915	Synonymous SNV	Benign	c.4308T>C (p.Ser1436=)	rs1060915	Synonymous SNV	Benign
				c.2311T>C (p.Leu771=)	rs16940	Synonymous SNV	Benign	c.2311T>C (p.Leu771=)	rs16940	Synonymous SNV	Benign
				c.2082C>T (p.Ser694=)	rs1799949	Synonymous SNV	Benign	c.2082C>T (p.Ser694=)	rs1799949	Synonymous SNV	Benign
				c.4900A>G (p.Ser1634Gly)	rs1799966	Non-synonymous SNV	Benign	c.4900A>G (p.Ser1634Gly)	rs1799966	Non-synonymous SNV	Benign
				c.3548A>G (p.Lys1183Arg)	rs16942	Non-synonymous SNV	Benign	c.3548A>G (p.Lys1183Arg)	rs16942	Non-synonymous SNV	Benign
				c.3113A>G (p.Glu1038Gly)	rs16941	Non-synonymous SNV	Benign	c.3113A>G (p.Glu1038Gly)	rs16941	Non-synonymous SNV	Benign
				c.2612C>T (p.Pro871Leu)	rs799917	Non-synonymous SNV	Benign	c.2612C>T (p.Pro871Leu)	rs799917	Non-synonymous SNV	Benign
				BRCA2							
				c.3807T>C (p.Val1269=)	rs543304	Synonymous SNV	Likely benign	Elimination			
				c.4563A>G (p.Leu1521=)	rs206075	Synonymous SNV	Benign	c.4563A>G (p.Leu1521=)	rs206075	Synonymous SNV	Benign
				c.6513G>C (p.Val2171=)	rs206076	Synonymous SNV	Benign	c.6513G>C (p.Val2171=)	rs206076	Synonymous SNV	Benign
				c.7397T>C (p.Val2466Ala)	rs169547	Non-synonymous SNV	Benign	c.7397T>C (p.Val2466Ala)	rs169547	Non-synonymous SNV	Benign
				PALB2							
				c.3114-51T>A	rs249936	Intronic	Benign	c.3114-51T>A	rs249936	Intronic	Benign
				c.1676A>G (p.Gln559Arg)	rs152451	Non-synonymous SNV	Benign	c.1676A>G (p.Gln559Arg)	rs152451	Non-synonymous SNV	Benign
				No mutations				c.3351-53delT	rs35294437	Intronic	VUS
				No mutations				c.2586+58C>T	rs249954	Intronic	Benign
Zh1	AC	75	PR	***BRCA1***							
				c.4308T>C (p.Ser1436=)	rs1060915	Synonymous SNV	Benign	c.4308T>C (p.Ser1436=)	rs1060915	Synonymous SNV	Benign

				c.2311T>C (p.Leu771=)	rs16940	Synonymous SNV	Benign	c.2311T>C (p.Leu771=)	rs16940	Synonymous SNV	Benign
				c.2082C>T (p.Ser694=)	rs1799949	Synonymous SNV	Benign	c.2082C>T (p.Ser694=)	rs1799949	Synonymous SNV	Benign
				c.4900A>G (p.Ser1634Gly)	rs1799966	Non-synonymous SNV	Benign	c.4900A>G (p.Ser1634Gly)	rs1799966	Non-synonymous SNV	Benign
				c.3548A>G (p.Lys1183Arg)	rs16942	Non-synonymous SNV	Benign	c.3548A>G (p.Lys1183Arg)	rs16942	Non-synonymous SNV	Benign
				c.3113A>G (p.Glu1038Gly)	rs16941	Non-synonymous SNV	Benign	c.3113A>G (p.Glu1038Gly)	rs16941	Non-synonymous SNV	Benign
				c.2612C>T (p.Pro871Leu)	rs799917	Non-synonymous SNV	Benign	c.2612C>T (p.Pro871Leu)	rs799917	Non-synonymous SNV	Benign
				BRCA2							
				c.3396A>G (p.Lys1132=)	rs1801406	Synonymous SNV	Little Clinical Significance	c.3396A>G (p.Lys1132=)	rs1801406	Synonymous SNV	Little Clinical Significance
				c.4563A>G (p.Leu1521=)	rs206075	Synonymous SNV	Benign	c.4563A>G (p.Leu1521=)	rs206075	Synonymous SNV	Benign
				c.6513G>C (p.Val2171=)	rs206076	Synonymous SNV	Benign	c.6513G>C (p.Val2171=)	rs206076	Synonymous SNV	Benign
				c.7242A>G (p.Ser2414=)	rs1799955	Synonymous SNV	Little Clinical Significance	c.7242A>G (p.Ser2414=)	rs1799955	Synonymous SNV	Little Clinical Significance
				c.590C>G (p.Ser197Cys)	rs876659940	Non-synonymous SNV	VUS	Elimination			
				c.7397T>C (p.Val2466Ala)	rs169547	Non-synonymous SNV	Benign	c.7397T>C (p.Val2466Ala)	rs169547	Non-synonymous SNV	Benign
				PALB2							
				c.3114-51T>A	rs249936	Intronic	Benign	c.3114-51T>A	rs249936	Intronic	Benign
L1	AC	99	PR	***BRCA1***							
				c.1067A>G (p.Gln356Arg)	rs1799950	Non-synonymous SNV	VUS	c.1067A>G (p.Gln356Arg)	rs1799950	Non-synonymous SNV	VUS
				BRCA2							
				c.3396A>G (p.Lys1132=)	rs1801406	Synonymous SNV	Little Clinical Significance	c.3396A>G (p.Lys1132=)	rs1801406	Synonymous SNV	Little Clinical Significance
				c.4563A>G (p.Leu1521=)	rs206075	Synonymous SNV	Benign	c.4563A>G (p.Leu1521=)	rs206075	Synonymous SNV	Benign
				c.6513G>C (p.Val2171=)	rs206076	Synonymous SNV	Benign	c.6513G>C (p.Val2171=)	rs206076	Synonymous SNV	Benign
				c.1114A>C (p.Asn372His)	rs144848	Non-synonymous SNV	Little Clinical Significance	Elimination			
				c.7397T>C (p.Val2466Ala)	rs169547	Non-synonymous SNV	Benign	c.7397T>C (p.Val2466Ala)	rs169547	Non-synonymous SNV	Benign
				PALB2							
				c.3114-51T>A	rs249936	Intronic	Benign	c.3114-51T>A	rs249936	Intronic	Benign
M1	AC	87	PR	***BRCA1***							
				c.4308T>C (p.Ser1436=)	rs1060915	Synonymous SNV	Benign	No data#			
				c.2311T>C (p.Leu771=)	rs16940	Synonymous SNV	Benign				
				c.2082C>T (p.Ser694=)	rs1799949	Synonymous SNV	Benign				
				c.4900A>G (p.Ser1634Gly)	rs1799966	Non-synonymous SNV	Benign				
				c.3548A>G (p.Lys1183Arg)	rs16942	Non-synonymous SNV	Benign				
				c.3113A>G (p.Glu1038Gly)	rs16941	Non-synonymous SNV	Benign				
				c.2612C>T (p.Pro871Leu)	rs799917	Non-synonymous SNV	Benign				

				BRCA2							
				c.3396A>G (p.Lys1132=)	rs1801406	Synonymous SNV	Little Clinical Significance				
				c.4563A>G (p.Leu1521=)	rs206075	Synonymous SNV	Benign				
				c.6513G>C (p.Val2171=)	rs206076	Synonymous SNV	Benign	No data			
				c.7242A>G (p.Ser2414=)	rs1799955	Synonymous SNV	Little Clinical Significance				
				c.7397T>C (p.Val2466Ala)	rs169547	Non-synonymous SNV	Benign				
				PALB2							
				c.3114-51T>A	rs249936	Intronic	Benign	No data			
M2	AC	81	PR	***BRCA1***							
				c.4308T>C (p.Ser1436=)	rs1060915	Synonymous SNV	Benign				
				c.2311T>C (p.Leu771=)	rs16940	Synonymous SNV	Benign				
				c.2082C>T (p.Ser694=)	rs1799949	Synonymous SNV	Benign				
				c.4900A>G (p.Ser1634Gly)	rs1799966	Non-synonymous SNV	Benign	No data			
				c.3548A>G (p.Lys1183Arg)	rs16942	Non-synonymous SNV	Benign				
				c.3113A>G (p.Glu1038Gly)	rs16941	Non-synonymous SNV	Benign				
				c.2612C>T (p.Pro871Leu)	rs799917	Non-synonymous SNV	Benign				
				BRCA2							
				c.3396A>G (p.Lys1132=)	rs1801406	Synonymous SNV	Little Clinical Significance				
				c.4068G>A (p.Leu1356=)	rs28897724	Synonymous SNV	Benign				
				c.4563A>G (p.Leu1521=)	rs206075	Synonymous SNV	Benign				
				c.6513G>C (p.Val2171=)	rs206076	Synonymous SNV	Benign	No data			
				c.7242A>G (p.Ser2414=)	rs1799955	Synonymous SNV	Little Clinical Significance				
				c.7397T>C (p.Val2466Ala)	rs169547	Non-synonymous SNV	Benign				
				PALB2							
				c.3114-51T>A	rs249936	Intronic	Benign	No data			
N1	AC	57	PR	***BRCA1***							
				No mutations				No mutations			
				BRCA2							
				c.3396A>G (p.Lys1132=)	rs1801406	Synonymous SNV	Little Clinical Significance	c.3396A>G (p.Lys1132=)	rs1801406	Synonymous SNV	Little Clinical Significance
				c.4563A>G (p.Leu1521=)	rs206075	Synonymous SNV	Benign	c.4563A>G (p.Leu1521=)	rs206075	Synonymous SNV	Benign
				c.6513G>C (p.Val2171=)	rs206076	Synonymous SNV	Benign	c.6513G>C (p.Val2171=)	rs206076	Synonymous SNV	Benign

				c.7242A>G (p.Ser2414=)	rs1799955	Synonymous SNV	Little Clinical Significance	c.7242A>G (p.Ser2414=)	rs1799955	Synonymous SNV	Little Clinical Significance
				c.7397T>C (p.Val2466Ala)	rs169547	Non-synonymous SNV	Benign	c.7397T>C (p.Val2466Ala)	rs169547	Non-synonymous SNV	Benign
				c.10076A>G (p.Glu3359Gly)	rs80358389	Non-synonymous SNV	VUS	c.10076A>G (p.Glu3359Gly)	rs80358389	Non-synonymous SNV	VUS
				PALB2							
				c.3114-51T>A	rs249936	Intronic	Benign	c.3114-51T>A	rs249936	Intronic	Benign
				c.2586+58C>T	rs249954	Intronic	Benign	c.2586+58C>T	rs249954	Intronic	Benign
P1	AC	-30	P	***BRCA1***							
				c.4035delA (p.Glu1345fs)	rs80357711	Frameshift deletion	Pathogenic	No data			
				BRCA2							
				c.1114A>C (p.Asn372His)	rs144848	Non-synonymous SNV	Little Clinical Significance	No data			
				c.3396A>G (p.Lys1132=)	rs1801406	Synonymous SNV	Little Clinical Significance				
				c.4563A>G (p.Leu1521=)	rs206075	Synonymous SNV	Benign				
				c.6513G>C (p.Val2171=)	rs206076	Synonymous SNV	Benign				
				c.7242A>G (p.Ser2414=)	rs1799955	Synonymous SNV	Little Clinical Significance				
				c.7397T>C (p.Val2466Ala)	rs169547	Non-synonymous SNV	Benign				
				PALB2							
				c.3300T>G (p.Thr1100=)	rs45516100	Synonymous SNV	Benign	No data[#]			
				c.3114-51T>A	rs249936	Intronic	Benign				
				c.2993G>A (p.Gly998Glu)	rs45551636	Non-synonymous SNV	Benign				
				c.2586+58C>T	rs249954	Intronic	Benign				
				c.2014G>C (p.Glu672Gln)	rs45532440	Non-synonymous SNV	Benign				
				c.1676A>G (p.Gln559Arg)	rs152451	Non-synonymous SNV	Benign				
				c.212-58A>C	rs80291632	Intronic	Benign				
S1	AC	12	ST	***BRCA1***							
				c.4900A>G (p.Ser1634Gly)	rs1799966	Non-synonymous SNV	Benign	No data			
				c.4308T>C (p.Ser1436=)	rs1060915	Synonymous SNV	Benign				
				c.3548A>G (p.Lys1183Arg)	rs16942	Non-synonymous SNV	Benign				
				c.3113A>G (p.Glu1038Gly)	rs16941	Non-synonymous SNV	Benign				
				c.2612C>T (p.Pro871Leu)	rs799917	Non-synonymous SNV	Benign				
				c.2311T>C (p.Leu771=)	rs16940	Synonymous SNV	Benign				
				c.2082C>T (p.Ser694Ser)	rs1799949	Synonymous SNV	Benign				
				BRCA2							

				c.3396A>G (p.Lys1132=)	rs1801406	Synonymous SNV	Little Clinical Significance	No data			
				c.4563A>G (p.Leu1521=)	rs206075	Synonymous SNV	Benign				
				c.6513G>C (p.Val2171=)	rs206076	Synonymous SNV	Benign				
				c.7397T>C (p.Val2466Ala)	rs169547	Non-synonymous SNV	Benign				
				c.9976A>T (p.Lys3326*)	rs11571833	Stopgain	Benign				
				PALB2							
				c.3351-53delT	rs35294437 (rs397855890)*	Intronic	VUS	No data			
				c.3114-51T>A	rs249936	Intronic	Benign				
				c.2586+58C>T	rs249954	Intronic	Benign				
				c.1676A>G (p.Gln559Arg)	rs152451	Non-synonymous SNV	Benign				
B3	CAX	82	PR	***BRCA1***							
				No mutations				c.3396A>G (p.Lys1132=)	rs1801406	Synonymous SNV	Little Clinical Significance
								c.4563A>G (p.Leu1521=)	rs206075	Synonymous SNV	Benign
								c.6513G>C (p.Val2171=)	rs206076	Synonymous SNV	Benign
								c.7242A>G (p.Ser2414=)	rs1799955	Synonymous SNV	Little Clinical Significance
								c.1114A>C (p.Asn372His)	rs144848	Non-synonymous SNV	Little Clinical Significance
				BRCA2							
				c.3396A>G (p.Lys1132=)	rs1801406	Synonymous SNV	Little Clinical Significance	c.3396A>G (p.Lys1132=)	rs1801406	Synonymous SNV	Little Clinical Significance
				c.4563A>G (p.Leu1521=)	rs206075	Synonymous SNV	Benign	c.4563A>G (p.Leu1521=)	rs206075	Synonymous SNV	Benign
				c.6513G>C (p.Val2171=)	rs206076	Synonymous SNV	Benign	c.6513G>C (p.Val2171=)	rs206076	Synonymous SNV	Benign
				c.7242A>G (p.Ser2414=)	rs1799955	Synonymous SNV	Little Clinical Significance	c.7242A>G (p.Ser2414=)	rs1799955	Synonymous SNV	Little Clinical Significance
				c.1114A>C (p.Asn372His)	rs144848	Non-synonymous SNV	Little Clinical Significance	Elimination			
				c.5455C>T (p.Pro1819Ser)	rs80358768	Non-synonymous SNV	Benign	c.5455C>T (p.Pro1819Ser)	rs80358768	Non-synonymous SNV	Benign
				c.5744C>T (p.Thr1915Met)	rs4987117	Non-synonymous SNV	Benign	Elimination			

				c.7397T>C (p.Val2466Ala)	rs169547	Non-synonymous SNV	Benign	c.7397T>C (p.Val2466Ala)	rs169547	Non-synonymous SNV	Benign
				PALB2							
				c.3351-53delT	rs35294437	Intronic	VUS	c.3351-53delT	rs35294437	Intronic	VUS
				c.3114-51T>A	rs249936	Intronic	Benign	c.3114-51T>A	rs249936	Intronic	Benign
				c.2586+58C>T	rs249954	Intronic	Benign	c.2586+58C>T	rs249954	Intronic	Benign
				c.1676A>G (p.Gln559Arg)	rs152451	Non-synonymous SNV	Benign	c.1676A>G (p.Gln559Arg)	rs152451	Non-synonymous SNV	Benign
V1	CAX	73	PR	***BRCA1***							
				c.1067A>G (p.Gln356Arg)	rs1799950	Non-synonymous SNV	VUS	c.1067A>G (p.Gln356Arg)	rs1799950	Non-synonymous SNV	VUS
				c.2612C>T (p.Pro871Leu)	rs799917	Non-synonymous SNV	Benign	c.2612C>T (p.Pro871Leu)	rs799917	Non-synonymous SNV	Benign
				BRCA2							
				c.3396A>G (p.Lys1132=)	rs1801406	Synonymous SNV	Little Clinical Significance	c.3396A>G (p.Lys1132=)	rs1801406	Synonymous SNV	Little Clinical Significance
				c.4563A>G (p.Leu1521=)	rs206075	Synonymous SNV	Benign	c.4563A>G (p.Leu1521=)	rs206075	Synonymous SNV	Benign
				c.6513G>C (p.Val2171=)	rs206076	Synonymous SNV	Benign	c.6513G>C (p.Val2171=)	rs206076	Synonymous SNV	Benign
				c.1114A>C (p.Asn372His)	rs144848	Non-synonymous SNV	Little Clinical Significance	Elimination			
				c.4258G>T (p.Asp1420Tyr)	rs28897727	Non-synonymous SNV	Benign	c.4258G>T (p.Asp1420Tyr)	rs28897727	Non-synonymous SNV	Benign
				c.7397T>C (p.Val2466Ala)	rs169547	Non-synonymous SNV	Benign	c.7397T>C (p.Val2466Ala)	rs169547	Non-synonymous SNV	Benign
				PALB2							
				c.3114-51T>A	rs249936	Intronic	Benign	c.3114-51T>A	rs249936	Intronic	Benign
G1	CAX	7	ST	***BRCA1***							
				c.2612C>T (p.Pro871Leu)	rs799917	Non-synonymous SNV	Benign	c.2612C>T (p.Pro871Leu)	rs799917	Non-synonymous SNV	Benign
				c.3113A>G (p.Glu1038Gly)	rs16941	Non-synonymous SNV	Benign	c.3113A>G (p.Glu1038Gly)	rs16941	Non-synonymous SNV	Benign
				c.4900A>G (p.Ser1634Gly)	rs1799966	Non-synonymous SNV	Benign	c.4900A>G (p.Ser1634Gly)	rs1799966	Non-synonymous SNV	Benign
				c.4946T>C (p.Met1649Thr)	rs4986854	Non-synonymous SNV	Benign	c.4946T>C (p.Met1649Thr)	rs4986854	Non-synonymous SNV	Benign
				c.3548A>G (p.Lys1183Arg)	rs16942	Non-synonymous SNV	Benign	c.3548A>G (p.Lys1183Arg)	rs16942	Non-synonymous SNV	Benign
				c.2082C>T (p.Ser694=)	rs1799949	Synonymous SNV	Benign	c.2082C>T (p.Ser694=)	rs1799949	Synonymous SNV	Benign
				c.4308T>C (p.Ser1436=)	rs1060915	Synonymous SNV	Benign	c.4308T>C (p.Ser1436=)	rs1060915	Synonymous SNV	Benign
				c.2311T>C (p.Leu771=)	rs16940	Synonymous SNV	Benign	c.2311T>C (p.Leu771=)	rs16940	Synonymous SNV	Benign
				BRCA2							
				c.3396A>G (p.Lys1132=)	rs1801406	Synonymous SNV	Little Clinical Significance	c.3396A>G (p.Lys1132=)	rs1801406	Synonymous SNV	Little Clinical Significance
				c.4563A>G (p.Leu1521=)	rs206075	Synonymous SNV	Benign	c.4563A>G (p.Leu1521=)	rs206075	Synonymous SNV	Benign
				c.6513G>C (p.Val2171=)	rs206076	Synonymous SNV	Benign	c.6513G>C (p.Val2171=)	rs206076	Synonymous SNV	Benign
				c.7242A>G (p.Ser2414=)	rs1799955	Synonymous SNV	Little Clinical Significance	c.7242A>G (p.Ser2414=)	rs1799955	Synonymous SNV	Little Clinical Significance
				c.7397T>C (p.Val2466Ala)	rs169547	Non-synonymous SNV	Benign	c.7397T>C (p.Val2466Ala)	rs169547	Non-synonymous SNV	Benign

				PALB2							
				c.3114-51T>A	rs249936	Intronic	Benign	c.3114-51T>A	rs249936	Intronic	Benign
				c.2586+58C>T	rs249954	Intronic	Benign	c.2586+58C>T	rs249954	Intronic	Benign
D2	CAX	89	PR	***BRCA1***							
				c.2612C>T (p.Pro871Leu)	rs799917	Non-synonymous SNV	Benign	No data			
				c.4900A>G (p.Ser1634Gly)	rs1799966	Non-synonymous SNV	Benign				
				c.3548A>G (p.Lys1183Arg)	rs16942	Non-synonymous SNV	Benign				
				c.3113A>G (p.Glu1038Gly)	rs16941	Non-synonymous SNV	Benign				
				c.2082C>T (p.Ser694=)	rs1799949	Synonymous SNV	Benign				
				c.4308T>C (p.Ser1436=)	rs1060915	Synonymous SNV	Benign				
				c.2311T>C (p.Leu771=)	rs16940	Synonymous SNV	Benign				
				BRCA2							
				c.3396A>G (p.Lys1132=)	rs1801406	Synonymous SNV	Little Clinical Significance	No data			
				c.4563A>G (p.Leu1521=)	rs206075	Synonymous SNV	Benign				
				c.6513G>C (p.Val2171=)	rs206076	Synonymous SNV	Benign				
				c.7242A>G (p.Ser2414=)	rs1799955	Synonymous SNV	Little Clinical Significance				
				c.6577G>C (p.Glu2193Gln)	-	Non-synonymous SNV	VUS				
				c.7397T>C (p.Val2466Ala)	rs169547	Non-synonymous SNV	Benign				
				PALB2							
				c.3114-51T>A	rs249936	Intronic	Benign	No data			
				c.2586+58C>T	rs249954	Intronic	Benign				
I1	CAX	100	CR	***BRCA1***							
				c.4308T>C (p.Ser1436=)	rs1060915	Synonymous SNV	Benign	c.4308T>C (p.Ser1436=)	rs1060915	Synonymous SNV	Benign
				c.2311T>C (p.Leu771=)	rs16940	Synonymous SNV	Benign	c.2311T>C (p.Leu771=)	rs16940	Synonymous SNV	Benign
				c.2082C>T (p.Ser694=)	rs1799949	Synonymous SNV	Benign	c.2082C>T (p.Ser694=)	rs1799949	Synonymous SNV	Benign
				c.4900A>G (p.Ser1634Gly)	rs1799966	Non-synonymous SNV	Benign	c.4900A>G (p.Ser1634Gly)	rs1799966	Non-synonymous SNV	Benign
				c.3548A>G (p.Lys1183Arg)	rs16942	Non-synonymous SNV	Benign	c.3548A>G (p.Lys1183Arg)	rs16942	Non-synonymous SNV	Benign
				c.3113A>G (p.Glu1038Gly)	rs16941	Non-synonymous SNV	Benign	c.3113A>G (p.Glu1038Gly)	rs16941	Non-synonymous SNV	Benign
				c.2612C>T (p.Pro871Leu)	rs799917	Non-synonymous SNV	Benign	c.2612C>T (p.Pro871Leu)	rs799917	Non-synonymous SNV	Benign
				BRCA2							
				c.4563A>G (p.Leu1521=)	rs206075	Synonymous SNV	Benign	c.4563A>G (p.Leu1521=)	rs206075	Synonymous SNV	Benign
				c.6513G>C (p.Val2171=)	rs206076	Synonymous SNV	Benign	c.6513G>C (p.Val2171=)	rs206076	Synonymous SNV	Benign
				c.125A>G (p.Tyr42Cys)	rs4987046	Non-synonymous SNV	Benign	c.125A>G (p.Tyr42Cys)	rs4987046	Non-synonymous SNV	Benign
				c.7397T>C (p.Val2466Ala)	rs169547	Non-synonymous SNV	Benign	c.7397T>C (p.Val2466Ala)	rs169547	Non-synonymous SNV	Benign
				PALB2							
				c.3114-51T>A	rs249936	Intronic	Benign	Elimination			
				c.2586+58C>T	rs249954	Intronic	Benign				
R1	CAX	-59	P	***BRCA1***							

				c.5019G>A (p.Met1652Ile)	rs1799967	Non-synonymous SNV	Benign	Elimination			
				BRCA2							
				c.-26G>A	rs1799943	5 prime UTR	VUS	Elimination			
				c.3396A>G (p.Lys1132=)	rs1801406	Synonymous SNV	Little Clinical Significance				
				c.4563A>G (p.Leu1521=)	rs206075	Synonymous SNV	Benign				
				c.6513G>C (p.Val2171=)	rs206076	Synonymous SNV	Benign				
				c.7242A>G (p.Ser2414=)	rs1799955	Synonymous SNV	Little Clinical Significance				
				c.7397T>C (p.Val2466Ala)	rs169547	Non-synonymous SNV	Benign				
				PALB2							
				c.3114-51T>A	rs249936	Intronic	Benign	c.3114-51T>A	rs249936	Intronic	Benign
S2	CAX	44	ST	***BRCA1***							
				c.5019G>A (p.Met1652Ile)	rs1799967	Non-synonymous SNV	Benign	c.5019G>A (p.Met1652Ile)	rs1799967	Non-synonymous SNV	Benign
				c.4900A>G (p.Ser1634Gly)	rs1799966	Non-synonymous SNV	Benign	c.4900A>G (p.Ser1634Gly)	rs1799966	Non-synonymous SNV	Benign
				c.4308T>C (p.Ser1436=)	rs1060915	Synonymous SNV	Benign	c.4308T>C (p.Ser1436=)	rs1060915	Synonymous SNV	Benign
				c.3548A>G (p.Lys1183Arg)	rs16942	Non-synonymous SNV	Benign	c.3548A>G (p.Lys1183Arg)	rs16942	Non-synonymous SNV	Benign
				c.3113A>G (p.Glu1038Gly)	rs16941	Non-synonymous SNV	Benign	c.3113A>G (p.Glu1038Gly)	rs16941	Non-synonymous SNV	Benign
				c.2612C>T (p.Pro871Leu)	rs799917	Non-synonymous SNV	Benign	c.2612C>T (p.Pro871Leu)	rs799917	Non-synonymous SNV	Benign
				c.2311T>C (p.Leu771=)	rs16940	Synonymous SNV	Benign	c.2311T>C (p.Leu771=)	rs16940	Synonymous SNV	Benign
				c.2082C>T (p.Ser694=)	rs1799949	Synonymous SNV	Benign	c.2082C>T (p.Ser694=)	rs1799949	Synonymous SNV	Benign
				BRCA2							
				No mutations				c.3807T>C (p.Val1269=)	rs543304	Synonymous SNV	Likely benign
				c.4563A>G (p.Leu1521=)	rs206075	Synonymous SNV	Benign	c.4563A>G (p.Leu1521=)	rs206075	Synonymous SNV	Benign
				c.6513G>C (p.Val2171=)	rs206076	Synonymous SNV	Benign	c.6513G>C (p.Val2171=)	rs206076	Synonymous SNV	Benign
				c.7397T>C (p.Val2466Ala)	rs169547	Non-synonymous SNV	Benign	c.7397T>C (p.Val2466Ala)	rs169547	Non-synonymous SNV	Benign
				PALB2							
				c.3114-51T>A	rs249936	Intronic	Benign	c.3114-51T>A	rs249936	Intronic	Benign
				c.2586+58C>T	rs249954	Intronic	Benign	c.2586+58C>T	rs249954	Intronic	Benign
S3	CAX	63	PR	***BRCA1***							
				No mutations				No mutations			
				BRCA2							
				c.-26G>A	rs1799943	5 prime UTR	VUS	Elimination			
				c.3396A>G (p.Lys1132=)	rs1801406	Synonymous SNV	Little Clinical Significance				
				c.4563A>G (p.Leu1521=)	rs206075	Synonymous SNV	Benign				
				c.6513G>C (p.Val2171=)	rs206076	Synonymous SNV	Benign				
				c.7397T>C (p.Val2466Ala)	rs169547	Non-synonymous SNV	Benign				

				PALB2							
				c.3114-51T>A	rs249936	Intronic	Benign	c.3114-51T>A	rs249936	Intronic	Benign
Kh1	CAX	81	PR	***BRCA1***							
				No mutations				No mutations			
				BRCA2							
				c.1114A>C (p.Asn372His)	rs144848	Non-synonymous SNV	Little Clinical Significance	Elimination			
				c.4563A>G (p.Leu1521=)	rs206075	Synonymous SNV	Benign				
				c.6513G>C (p.Val2171=)	rs206076	Synonymous SNV	Benign				
				c.7397T>C (p.Val2466Ala)	rs169547	Non-synonymous SNV	Benign				
				PALB2							
				c.3114-51T>A	rs249936	Intronic	Benign	c.3114-51T>A	rs249936	Intronic	Benign
				c.2586+58C>T	rs249954	Intronic	Benign	c.2586+58C>T	rs249954	Intronic	Benign
Ch1	CAX	60	PR	***BRCA1***							
				No mutations				No mutations			
				BRCA2							
				c.865A>C (p.Asn289His)	rs766173	Non-synonymous SNV	Benign	Elimination			
				c.1365A>G (p.Ser455=)	rs1801439	Synonymous SNV	Benign	c.1365A>G (p.Ser455=)	rs1801439	Synonymous SNV	Benign
				c.2229T>C (p.His743=)	rs1801499	Synonymous SNV	Benign	c.2229T>C (p.His743=)	rs1801499	Synonymous SNV	Benign
				c.2971A>G (p.Asn991Asp)	rs1799944	Non-synonymous SNV	Benign	c.2971A>G (p.Asn991Asp)	rs1799944	Non-synonymous SNV	Benign
				c.4563A>G (p.Leu1521=)	rs206075	Synonymous SNV	Benign	c.4563A>G (p.Leu1521=)	rs206075	Synonymous SNV	Benign
				c.6513G>C (p.Val2171=)	rs206076	Synonymous SNV	Benign	c.6513G>C (p.Val2171=)	rs206076	Synonymous SNV	Benign
				c.7397T>C (p.Val2466Ala)	rs169547	Non-synonymous SNV	Benign	c.7397T>C (p.Val2466Ala)	rs169547	Non-synonymous SNV	Benign
				c.8673_8674delAA (p.Thr2891fs)	rs80359724	Frameshift deletion	Pathogenic	c.8673_8674delAA (p.Thr2891fs)	rs80359724	Frameshift deletion	Pathogenic
				PALB2							
				c.3114-51T>A	rs249936	Intronic	Benign	c.3114-51T>A	rs249936	Intronic	Benign
				c.2586+58C>T	rs249954	Intronic	Benign	c.2586+58C>T	rs249954	Intronic	Benign
Sh1	CAX	43	ST	***BRCA1***							
				c.1067A>G (p.Gln356Arg)	rs1799950	Non-synonymous SNV	VUS	c.1067A>G (p.Gln356Arg)	rs1799950	Non-synonymous SNV	VUS
				BRCA2							
				c.1114A>C (p.Asn372His)	rs144848	Non-synonymous SNV	Little Clinical Significance	Elimination			
				c.4563A>G (p.Leu1521=)	rs206075	Synonymous SNV	Benign	c.4563A>G (p.Leu1521=)	rs206075	Synonymous SNV	Benign
				c.6513G>C (p.Val2171=)	rs206076	Synonymous SNV	Benign	c.6513G>C (p.Val2171=)	rs206076	Synonymous SNV	Benign
				c.7397T>C (p.Val2466Ala)	rs169547	Non-synonymous SNV	Benign	c.7397T>C (p.Val2466Ala)	rs169547	Non-synonymous SNV	Benign
				c.10234A>G (p.Ile3412Val)	rs1801426	Non-synonymous SNV	Benign	c.10234A>G (p.Ile3412Val)	rs1801426	Non-synonymous SNV	Benign
				PALB2							
				c.3351-53delT	rs35294437	Intronic	VUS	c.3351-53delT	rs35294437	Intronic	VUS
				c.3114-51T>A	rs249936	Intronic	Benign	c.3114-51T>A	rs249936	Intronic	Benign
				c.2586+58C>T	rs249954	Intronic	Benign	c.2586+58C>T	rs249954	Intronic	Benign

				c.1676A>G (p.Gln559Arg)	rs152451	Non-synonymous SNV	Benign	c.1676A>G (p.Gln559Arg)	rs152451	Non-synonymous SNV	Benign
A1	Taxotere	100	CR	***BRCA1***							
				c.4900A>G (p.Ser1634Gly)	rs1799966	Non-synonymous SNV	Benign	Elimination			
				c.2311T>C (p.Leu771=)	rs16940	Synonymous SNV	Benign	c.2311T>C (p.Leu771=)	rs16940	Synonymous SNV	Benign
				c.2612C>T (p.Pro871Leu)	rs799917	Non-synonymous SNV	Benign	c.2612C>T (p.Pro871Leu)	rs799917	Non-synonymous SNV	Benign
				c.3113A>G (p.Glu1038Gly)	rs16941	Non-synonymous SNV	Benign	c.3113A>G (p.Glu1038Gly)	rs16941	Non-synonymous SNV	Benign
				c.3548A>G (p.Lys1183Arg)	rs16942	Non-synonymous SNV	Benign	c.3548A>G (p.Lys1183Arg)	rs16942	Non-synonymous SNV	Benign
				c.2082C>T (p.Ser694=)	rs1799949	Synonymous SNV	Benign	c.2082C>T (p.Ser694=)	rs1799949	Synonymous SNV	Benign
				No mutations				c.2609C>G (p.Ala870Gly)	rs1060502324	Non-synonymous SNV	VUS
								c.4837A>G (p.Ser1613Gly)	rs1799966	Non-synonymous SNV	Benign
								c.999T>C (p.Ser333=)	-	Synonymous SNV	Benign
				c.4308T>C (p.Ser1436=)	rs1060915	Synonymous SNV	Benign	Elimination			
				c.2470_2471insTTCCGATC TTAGTCC (p.Pro 824delinsLPILVP)	-	Non-frameshift insertion	VUS				
				BRCA2							
				c.1114A>C (p.Asn372His)	rs144848	Non-synonymous SNV	Little Clinical Significance	c.1114A>C (p.Asn372His)	rs144848	Non-synonymous SNV	Little Clinical Significance
				No mutations				c.3823_3824insAGCAGTTC C p.Ieu1275delinsKQFL	-	Non-frameshift insertion	VUS
				c.4563A>G (p.Leu1521=)	rs206075	Synonymous SNV	Benign	c.4563A>G (p.Leu1521=)	rs206075	Synonymous SNV	Benign
				c.6513G>C (p.Val2171=)	rs206076	Synonymous SNV	Benign	c.6513G>C (p.Val2171=)	rs206076	Synonymous SNV	Benign
				c.7397T>C (p.Val2466Ala)	rs169547	Non-synonymous SNV	Benign	c.7397T>C (p.Val2466Ala)	rs169547	Non-synonymous SNV	Benign
				No mutations				c.8878C>G (p.Gln2960Glu)	-	Non-synonymous SNV	VUS
								c.8881_8884del (p.Gly2961fs)	-	Frameshift deletion	VUS
								c.8885T>A	-	Stopgain	VUS
								c.1295A>T (p.Glu432Val)	-	Non-synonymous SNV	VUS
								c.1305A>G (p.Arg435=)	-	Synonymous SNV	VUS
								c.1309_1310insAAATC	-	Frameshift insertion	VUS
				PALB2							
				c.3114-51T>A	rs249936	Intronic	Benign	Elimination			
				c.2552delA (p.Asn851fs)	-	Frameshift deletion	VUS				
B4	ACT	83	PR	***BRCA1***							
				c.2609C>G (p.Ala870Gly)	rs1060502324	Non-synonymous SNV	VUS	c.2609C>G (p.Ala870Gly)	rs1060502324	Non-synonymous SNV	VUS
				c.2608G>A (p.Ala870Thr)	rs753256448	Non-synonymous SNV	VUS	c.2608G>A (p.Ala870Thr)	rs753256448	Non-synonymous SNV	VUS
				c.4807_4821del (p.Pro1603_Val1607del)	rs80359888	Non-frameshift deletion	VUS	Elimination			
				BRCA2							
				c.7397T>C (p.Val2466Ala)	rs169547	Non-synonymous SNV	Benign	c.7397T>C (p.Val2466Ala)	rs169547	Non-synonymous SNV	Benign
				c.6513G>C (p.Val2171=)	rs206076	Synonymous SNV	Benign	c.6513G>C (p.Val2171=)	rs206076	Synonymous SNV	Benign
				c.4563A>G (p.Leu1521=)	rs206075	Synonymous SNV	Benign	c.4563A>G (p.Leu1521=)	rs206075	Synonymous SNV	Benign
				c.7108_7109insCAT	-	Stopgain	VUS	c.7108_7109insCAT	-	Stopgain	VUS

				(p.K2370delinsTX)				(p.K2370delinsTX)			
				c.7110_7111insATATGTGG G (p.Lys2370delinsKICG)	-	Non-frameshift insertion	VUS	c.7110_7111insATATGTGG G (p.Lys2370delinsKICG)	-	Non-frameshift insertion	VUS
				c.4288_4289insGGAACTGA GT (p.Thr1430_1431Ala_delinsR NX)	-	Stopgain	VUS	c.4288_4289insGGAACTG AGT (p.Thr1430_1431Ala_delins RNX)	-	Stopgain	VUS
				c.3807T>C (p.Val1269=)	rs543304	Synonymous SNV	Likely benign	c.3807T>C (p.Val1269=)	rs543304	Synonymous SNV	Likely benign
				No mutations				c.9090delA (p.T3030fs)	rs397507420	Frameshift deletion	Pathoge nic
								c.9090dupA (p.Thr3030fs)	-	Frameshift duplication	VUS
								c.8878C>T p.(Gln2960Ter)	-	Non-synonymous SNV	Pathoge nic
								c.8881_8884del (p.Gly2961fs)	-	Frameshift deletion	VUS
								c.8885T>A	-	Stopgain	VUS
				PALB2							
				c.3114-51T>A	rs249936	Intronic	Benign	c.3114-51T>A	rs249936	Intronic	Benign
				c.2586+58C>T	rs249954	Intronic	Benign	c.2586+58C>T	rs249954	Intronic	Benign
				c.2552delA (p.Asn851fs)	-	Frameshift deletion	VUS	Elimination			
				No mutations				c.3351-53delT	rs35294437 (rs780210723) **	Intronic	VUS
V2	Taxotere	68	PR	***BRCA1***							
				c.4900A>G (p.Ser1634Gly)	rs1799966	Non-synonymous SNV	Benign	c.4900A>G (p.Ser1634Gly)	rs1799966	Non-synonymous SNV	Benign
				c.2311T>C (p.Leu771=)	rs16940	Synonymous SNV	Benign	c.2311T>C (p.Leu771=)	rs16940	Synonymous SNV	Benign
				c.2612C>T (p.Pro871Leu)	rs799917	Non-synonymous SNV	Benign	c.2612C>T (p.Pro871Leu)	rs799917	Non-synonymous SNV	Benign
				c.2470_2471insTTCCGATC TTAGTCC (p.Pro 824delinsLPILVP)	-	Non-frameshift insertion	VUS	c.2470_2471insTTCCGATC TTAGTCC (p.Pro 824delinsLPILVP)	-	Non-frameshift insertion	VUS
				c.3548A>G (p.Lys1183Arg)	rs16942	Non-synonymous SNV	Benign	c.3548A>G (p.Lys1183Arg)	rs16942	Non-synonymous SNV	Benign
				c.4308T>C (p.Ser1436=)	rs1060915	Synonymous SNV	Benign	c.4308T>C (p.Ser1436=)	rs1060915	Synonymous SNV	Benign
				c.3113A>G (p.Glu1038Gly)	rs16941	Non-synonymous SNV	Benign	c.3113A>G (p.Glu1038Gly)	rs16941	Non-synonymous SNV	Benign
				c.2082C>T (p.Ser694=)	rs1799949	Synonymous SNV	Benign	c.2082C>T (p.Ser694=)	rs1799949	Synonymous SNV	Benign
				c.4807_4821del (p.Pro1603_Val1607del)	rs80359888	Non-frameshift deletion	VUS	Elimination			
				BRCA2							
				c.7397T>C (p.Val2466Ala)	rs169547	Non-synonymous SNV	Benign	c.7397T>C (p.Val2466Ala)	rs169547	Non-synonymous SNV	Benign
				c.6513G>C (p.Val2171=)	rs206076	Synonymous SNV	Benign	c.6513G>C (p.Val2171=)	rs206076	Synonymous SNV	Benign
				c.8878C>T p.(Gln2960Ter)	-	Non-synonymous SNV	Pathoge nic	c.4563A>G (p.Leu1521=)	rs206075	Synonymous SNV	Benign
				c.1114A>C (p.Asn372His)	rs144848	Non-synonymous SNV	Little Clinical Significance	c.1114A>C (p.Asn372His)	rs144848	Non-synonymous SNV	Little Clinical Significance

				c.3396A>G (p.Lys1132=)	rs1801406	Synonymous SNV	Little Clinical Significance	c.3396A>G (p.Lys1132=)	rs1801406	Synonymous SNV	Little Clinical Significance
				c.7242A>G (p.Ser2414=)	rs1799955	Synonymous SNV	Little Clinical Significance	c.7242A>G (p.Ser2414=)	rs1799955	Synonymous SNV	Little Clinical Significance
				No mutations				c.9090delA (p.T3030fs)	rs397507420	Frameshift deletion	Pathogenic
								c.9090dupA (p.Thr3030fs)	-	Frameshift duplication	VUS
				c.4563A>G (p.Leu1521=)	rs206075	Synonymous SNV	Benign	Elimination			
				c.3823_3824insAGCAGTTCC (p.Ile1275delinsKQFL)	-	Frameshift insertion	VUS				
				c.3824_3825del p.(Ile1275ArgfsTer4)	-	Frameshift deletion	Pathogenic				
				c.8881_8884del (p.Gly2961fs)	-	Frameshift deletion	VUS				
				c.8885T>A	-	Stopgain	VUS				
				PALB2							
				c.3114-51T>A	rs249936	Intronic	Benign	c.3114-51T>A	rs249936	Intronic	Benign
				No mutations				c.3351-53delT	rs35294437	Intronic	VUS
D3	AT	94	PR	***BRCA1***							
				No mutations				No mutations			
				BRCA2							
				c.7397T>C (p.Val2466Ala)	rs169547	Non-synonymous SNV	Benign	c.7397T>C (p.Val2466Ala)	rs169547	Non-synonymous SNV	Benign
				c.6513G>C (p.Val2171=)	rs206076	Synonymous SNV	Benign	c.6513G>C (p.Val2171=)	rs206076	Synonymous SNV	Benign
				c.4563A>G (p.Leu1521=)	rs206075	Synonymous SNV	Benign	c.4563A>G (p.Leu1521=)	rs206075	Synonymous SNV	Benign
				c.1114A>C (p.Asn372His)	rs144848	Non-synonymous SNV	Little Clinical Significance	c.1114A>C (p.Asn372His)	rs144848	Non-synonymous SNV	Little Clinical Significance
				c.3396A>G (p.Lys1132=)	rs1801406	Synonymous SNV	Little Clinical Significance	c.3396A>G (p.Lys1132=)	rs1801406	Synonymous SNV	Little Clinical Significance
				c.3823_3824insAGCAGTTCC (p.Ile1275delinsKQFL)	-	Frameshift insertion	VUS	c.3823_3824insAGCAGTTCC (p.Ile1275delinsKQFL)	-	Frameshift insertion	VUS
				c.3824delT (p.Ile 1275fs)	-	Frameshift deletion	VUS	c.3824delT (p.Ile 1275fs)	-	Frameshift deletion	VUS
				c.7242A>G (p.Ser2414=)	rs1799955	Synonymous SNV	Little Clinical Significance	c.7242A>G (p.Ser2414=)	rs1799955	Synonymous SNV	Little Clinical Significance
				No mutations				c.5744C>T (p.Thr1915Met)	rs4987117	Non-synonymous SNV	Benign
								c.8878C>G (p.Gln2960Glu)	-	Non-synonymous SNV	VUS
								c.8881_8884del (p.Gly2961fs)	-	Frameshift deletion	VUS

								c.8885T>A	-	Stopgain	VUS
								c.9090delA (p.T3030fs)	rs397507420	Frameshift deletion	Pathogenic
								c.9090dupA (p.Thr3030fs)	-	Frameshift duplication	VUS
				PALB2							
				c.3114-51T>A	rs249936	Intronic	Benign	Elimination			
				No mutations				c.2586+58C>T	rs249954	Intronic	Benign
D4	Taxotere	86	PR	***BRCA1***							
				c.1067A>G (p.Gln356Arg)	rs1799950	Non-synonymous SNV	VUS	c.1067A>G (p.Gln356Arg)	rs1799950	Non-synonymous SNV	VUS
				BRCA2							
				c.7397T>C (p.Val2466Ala)	rs169547	Non-synonymous SNV	Benign	c.7397T>C (p.Val2466Ala)	rs169547	Non-synonymous SNV	Benign
				c.6513G>C (p.Val2171=)	rs206076	Synonymous SNV	Benign	c.6513G>C (p.Val2171=)	rs206076	Synonymous SNV	Benign
				c.4563A>G (p.Leu1521=)	rs206075	Synonymous SNV	Benign	c.4563A>G (p.Leu1521=)	rs206075	Synonymous SNV	Benign
				c.3807T>C (p.Val1269=)	rs543304	Synonymous SNV	Likely benign	c.3807T>C (p.Val1269=)	rs543304	Synonymous SNV	Likely benign
				c.3823_3824insAGCAGTTCC (p.Ile1275delinsKQFL)	-	Frameshift insertion	VUS	c.3824_3825del p.(Ile1275ArgfsTer4)	-	Frameshift deletion	Pathogenic
				c.3824_3825del p.(Ile1275ArgfsTer4)	-	Frameshift deletion	Pathogenic	c.3823_3824insAGCAGTTCC (p.Ile1275delinsKQFL)	-	Frameshift insertion	VUS
				c.9090delA (p.T3030fs)	rs397507420	Frameshift deletion	Pathogenic	Elimination			
				c.9090dupA (p.Thr3030fs)	-	Frameshift duplication	VUS				
				PALB2							
				c.3351-53delT	rs35294437	Intronic	VUS	c.3351-53delT	rs35294437	Intronic	VUS
				c.3114-51T>A	rs249936	Intronic	Benign	c.3114-51T>A	rs249936	Intronic	Benign
				c.2586+58C>T	rs249954	Intronic	Benign	c.2586+58C>T	rs249954	Intronic	Benign
				c.1676A>G (p.Gln559Arg)	rs152451	Non-synonymous SNV	Benign	c.1676A>G (p.Gln559Arg)	rs152451	Non-synonymous SNV	Benign
				c.1675_1676insGAGTGAAAGGTAAATCAAGATGTGTGCTCTTCCGACTCC (p.Q559delinsRVKGKSRCVLFRLQ)	-	Non-frameshift insertion	VUS	c.1675_1676insGAGTGAAAGGTAAATCAAGATGTGTGCTCTTCCGACTCC (p.Q559delinsRVKGKSRCVLFRLQ)	-	Non-frameshift insertion	VUS
Zh2	AT	0	ST	***BRCA1***							
				c.4900A>G (p.Ser1634Gly)	rs1799966	Non-synonymous SNV	Benign	c.4900A>G (p.Ser1634Gly)	rs1799966	Non-synonymous SNV	Benign
				c.2311T>C (p.Leu771=)	rs16940	Synonymous SNV	Benign	c.2311T>C (p.Leu771=)	rs16940	Synonymous SNV	Benign
				c.2612C>T (p.Pro871Leu)	rs799917	Non-synonymous SNV	Benign	c.2612C>T (p.Pro871Leu)	rs799917	Non-synonymous SNV	Benign
				c.2470_2471insTTCCGATCTTAGTCC (p.Pro 824delinsLPILVP)	-	Non-frameshift insertion	VUS	c.2470_2471insTTCCGATCTTAGTCC (p.Pro 824delinsLPILVP)	-	Non-frameshift insertion	VUS
				c.3548A>G (p.Lys1183Arg)	rs16942	Non-synonymous SNV	Benign	c.3548A>G (p.Lys1183Arg)	rs16942	Non-synonymous SNV	Benign
				c.4308T>C (p.Ser1436=)	rs1060915	Synonymous SNV	Benign	c.4308T>C (p.Ser1436=)	rs1060915	Synonymous SNV	Benign
				c.3113A>G (p.Glu1038Gly)	rs16941	Non-synonymous SNV	Benign	c.3113A>G (p.Glu1038Gly)	rs16941	Non-synonymous SNV	Benign
				c.2082C>T (p.Ser694=)	rs1799949	Synonymous SNV	Benign	c.2082C>T (p.Ser694=)	rs1799949	Synonymous SNV	Benign
				c.1067A>G (p.Gln356Arg)	rs1799950	Non-synonymous SNV	VUS	c.1067A>G (p.Gln356Arg)	rs1799950	Non-synonymous SNV	VUS
				BRCA2							

				c.7397T>C (p.Val2466Ala)	rs169547	Non-synonymous SNV	Benign	c.7397T>C (p.Val2466Ala)	rs169547	Non-synonymous SNV	Benign
				c.6513G>C (p.Val2171=)	rs206076	Synonymous SNV	Benign	c.6513G>C (p.Val2171=)	rs206076	Synonymous SNV	Benign
				c.4563A>G (p.Leu1521=)	rs206075	Synonymous SNV	Benign	c.4563A>G (p.Leu1521=)	rs206075	Synonymous SNV	Benign
				c.1114A>C (p.Asn372His)	rs144848	Non-synonymous SNV	Little Clinical Significance	c.1114A>C (p.Asn372His)	rs144848	Non-synonymous SNV	Little Clinical Significance
				c.3823_3824insAGCAGTTCC (p.Ile1275delinsKQFL)	-	Frameshift insertion	VUS	Elimination			
				c.3824_3825del p.(Ile1275ArgfsTer4)	-	Frameshift deletion	Pathogenic				
				PALB2							
				c.3114-51T>A	rs249936	Intronic	Benign	c.3114-51T>A	rs249936	Intronic	Benign
				c.834A>T (p.Leu278=)	rs199919863	Synonymous SNV	Likely benign	c.834A>T (p.Leu278=)	rs199919863	Synonymous SNV	Likely benign
				c.833T>A (p.Leu278Gln)	rs200843485	Non-synonymous SNV	VUS	c.833T>A (p.Leu278Gln)	rs200843485	Non-synonymous SNV	VUS
				c.1706_1707del (p.Lys569fs)	rs1060502759	Frameshift deletion	Pathogenic	Elimination			
				c.2586+58C>T	rs249954	Intronic	Benign				
				No mutations				c.2587-38C>T	rs180177119	Intronic	Likely benign
K1	ACT	80	PR	***BRCA1***							
				c.4900A>G (p.Ser1634Gly)	rs1799966	Non-synonymous SNV	Benign	c.4900A>G (p.Ser1634Gly)	rs1799966	Non-synonymous SNV	Benign
				c.2311T>C (p.Leu771=)	rs16940	Synonymous SNV	Benign	c.2311T>C (p.Leu771=)	rs16940	Synonymous SNV	Benign
				c.2612C>T (p.Pro871Leu)	rs799917	Non-synonymous SNV	Benign	c.2612C>T (p.Pro871Leu)	rs799917	Non-synonymous SNV	Benign
				c.2470_2471insTTCCGATCTTAGTCC (p.Pro 824delinsLPILVP)	-	Non-frameshift insertion	VUS	c.2470_2471insTTCCGATCTTAGTCC (p.Pro 824delinsLPILVP)	-	Non-frameshift insertion	VUS
				c.3548A>G (p.Lys1183Arg)	rs16942	Non-synonymous SNV	Benign	c.3548A>G (p.Lys1183Arg)	rs16942	Non-synonymous SNV	Benign
				c.4308T>C (p.Ser1436=)	rs1060915	Synonymous SNV	Benign	c.4308T>C (p.Ser1436=)	rs1060915	Synonymous SNV	Benign
				c.3113A>G (p.Glu1038Gly)	rs16941	Non-synonymous SNV	Benign	c.3113A>G (p.Glu1038Gly)	rs16941	Non-synonymous SNV	Benign
				c.2082C>T (p.Ser694=)	rs1799949	Synonymous SNV	Benign	c.2082C>T (p.Ser694=)	rs1799949	Synonymous SNV	Benign
				BRCA2							
				c.7397T>C (p.Val2466Ala)	rs169547	Non-synonymous SNV	Benign	c.7397T>C (p.Val2466Ala)	rs169547	Non-synonymous SNV	Benign
				c.6513G>C (p.Val2171=)	rs206076	Synonymous SNV	Benign	c.6513G>C (p.Val2171=)	rs206076	Synonymous SNV	Benign
				c.8878C>T p.(Gln2960Ter)	-	Non-synonymous SNV	Pathogenic	c.8878C>T p.(Gln2960Ter)	-	Non-synonymous SNV	Pathogenic
				c.8881_8884del (p.Gly2961fs)	-	Frameshift deletion	VUS	c.8881_8884del (p.Gly2961fs)	-	Frameshift deletion	VUS
				c.8885T>A	-	Stopgain	VUS	c.8885T>A	-	Stopgain	VUS
				c.4563A>G (p.Leu1521=)	rs206075	Synonymous SNV	Benign	c.4563A>G (p.Leu1521=)	rs206075	Synonymous SNV	Benign
				c.1114A>C (p.Asn372His)	rs144848	Non-synonymous SNV	Little Clinical Significance	c.1114A>C (p.Asn372His)	rs144848	Non-synonymous SNV	Little Clinical Significance
				c.9090delA (p.T3030fs)	rs397507420	Frameshift deletion	Pathoge	c.9090delA (p.T3030fs)	rs397507420	Frameshift deletion	Pathoge

							nic				nic
				c.9090dupA (p.Thr3030fs)	-	Frameshift duplication	VUS	c.9090dupA (p.Thr3030fs)	-	Frameshift duplication	VUS
				No mutations				c.3823_3824insAGCAGTTCC (p.Ile1275delinsKQFL)	-	Frameshift insertion	VUS
								c.3824_3825del p.(Ile1275ArgfsTer4)	-	Frameshift deletion	Pathogenic
				PALB2							
				c.3114-51T>A	rs249936	Intronic	Benign	c.3114-51T>A	rs249936	Intronic	Benign
				c.2586+58C>T	rs249954	Intronic	Benign	c.2586+58C>T	rs249954	Intronic	Benign
				c.94C>G (p.Leu32Val)	rs151316635	Non-synonymous SNV	Conflicting interpretations of pathogenicity	c.94C>G (p.Leu32Val)	rs151316635	Non-synonymous SNV	Conflicting interpretations of pathogenicity
				c.3351-53delT	rs35294437	Intronic	VUS	Elimination			
L2	Taxotere	35	ST	***BRCA1***							
				c.4900A>G (p.Ser1634Gly)	rs1799966	Non-synonymous SNV	Benign	c.4900A>G (p.Ser1634Gly)	rs1799966	Non-synonymous SNV	Benign
				c.2311T>C (p.Leu771=)	rs16940	Synonymous SNV	Benign	c.2311T>C (p.Leu771=)	rs16940	Synonymous SNV	Benign
				c.2612C>T (p.Pro871Leu)	rs799917	Non-synonymous SNV	Benign	c.2612C>T (p.Pro871Leu)	rs799917	Non-synonymous SNV	Benign
				c.2470_2471insTTCCGATCTTAGTCC (p.Pro 824delinsLPILVP)	-	Non-frameshift insertion	VUS	c.2470 2471insTTCCGATCTTAGTCC (p.Pro 824delinsLPILVP)	-	Non-frameshift insertion	VUS
				c.3548A>G (p.Lys1183Arg)	rs16942	Non-synonymous SNV	Benign	c.3548A>G (p.Lys1183Arg)	rs16942	Non-synonymous SNV	Benign
				c.4308T>C (p.Ser1436=)	rs1060915	Synonymous SNV	Benign	c.4308T>C (p.Ser1436=)	rs1060915	Synonymous SNV	Benign
				c.3113A>G (p.Glu1038Gly)	rs16941	Non-synonymous SNV	Benign	c.3113A>G (p.Glu1038Gly)	rs16941	Non-synonymous SNV	Benign
				c.2082C>T (p.Ser694=)	rs1799949	Synonymous SNV	Benign	c.2082C>T (p.Ser694=)	rs1799949	Synonymous SNV	Benign
				BRCA2							
				c.3807T>C (p.Val1269=)	rs543304	Synonymous SNV	Likely benign	Elimination			
				c.4563A>G (p.Leu1521=)	rs206075	Synonymous SNV	Benign	c.4563A>G (p.Leu1521=)	rs206075	Synonymous SNV	Benign
				c.6513G>C (p.Val2171=)	rs206076	Synonymous SNV	Benign	c.6513G>C (p.Val2171=)	rs206076	Synonymous SNV	Benign
				c.7397T>C (p.Val2466Ala)	rs169547	Non-synonymous SNV	Benign	c.7397T>C (p.Val2466Ala)	rs169547	Non-synonymous SNV	Benign
				c.8878C>G (p.Gln2960Glu)	-	Non-synonymous SNV	VUS	c.8878C>G (p.Gln2960Glu)	-	Non-synonymous SNV	VUS
				c.8881_8884del (p.Gly2961fs)	-	Frameshift deletion	VUS	c.8881_8884del (p.Gly2961fs)	-	Frameshift deletion	VUS
				c.8885T>A	-	Stopgain	VUS	c.8885T>A	-	Stopgain	VUS
				PALB2							
				c.3114-51T>A	rs249936	Intronic	Benign	c.3114-51T>A	rs249936	Intronic	Benign
				c.2586+58C>T	rs249954	Intronic	Benign	c.2586+58C>T	rs249954	Intronic	Benign
				c.1676A>G (p.Gln559Arg)	rs152451	Non-synonymous SNV	Benign	c.1676A>G (p.Gln559Arg)	rs152451	Non-synonymous SNV	Benign
				c.1675_1676insGAGTGAAAGGTAAATCAAGATGTGTGCTCTTCCGACTCC (p.Q559delinsRVKGKSRCV	-	Non-frameshift insertion	VUS	Elimination			

				LFRLQ)							
				No mutations				c.3351-53delT	rs35294437	Intronic	VUS
R2	Taxotere	21	ST	***BRCA1***							
				c.4900A>G (p.Ser1634Gly)	rs1799966	Non-synonymous SNV	Benign				
				c.2311T>C (p.Leu771=)	rs16940	Synonymous SNV	Benign	Elimination			
				c.2612C>T (p.Pro871Leu)	rs799917	Non-synonymous SNV	Benign				
				c.3113A>G (p.Glu1038Gly)	rs16941	Non-synonymous SNV	Benign				
				c.3548A>G (p.Lys1183Arg)	rs16942	Non-synonymous SNV	Benign				
				c.4308T>C (p.Ser1436=)	rs1060915	Synonymous SNV	Benign				
				c.2470_2471insTTCCGATC TTAGTCC (p.Pro 824delinsLPILVP)	-	Non-frameshift insertion	VUS				
				No mutations				c.4807_4821del (p.Pro1603_Val1607del)	rs80359888	Non-frameshift deletion	VUS
				BRCA2							
				c.1114A>C (p.Asn372His)	rs144848	Non-synonymous SNV	Little Clinical Significance	c.1114A>C (p.Asn372His)	rs144848	Non-synonymous SNV	Little Clinical Significance
				c.3396A>G (p.Lys1132=)	rs1801406	Synonymous SNV	Little Clinical Significance	c.3396A>G (p.Lys1132=)	rs1801406	Synonymous SNV	Little Clinical Significance
				c.3823_3824insAGCAGTTC C (p.Ile1275delinsKQFL)	-	Frameshift insertion	VUS	c.3823_3824insAGCAGTTC C (p.Ile1275delinsKQFL)	-	Frameshift insertion	VUS
				c.4563A>G (p.Leu1521=)	rs206075	Synonymous SNV	Benign	c.4563A>G (p.Leu1521=)	rs206075	Synonymous SNV	Benign
				c.5744C>T (p.Thr1915Met)	rs4987117	Non-synonymous SNV	Benign	c.5744C>T (p.Thr1915Met)	rs4987117	Non-synonymous SNV	Benign
				c.3824delT (p.Ile 1275fs)	-	Frameshift deletion	VUS	c.3824delT (p.Ile 1275fs)	-	Frameshift deletion	VUS
				c.6513G>C (p.Val2171=)	rs206076	Synonymous SNV	Benign	c.6513G>C (p.Val2171=)	rs206076	Synonymous SNV	Benign
				c.7242A>G (p.Ser2414=)	rs1799955	Synonymous SNV	Little Clinical Significance	Elimination			
				c.7397T>C (p.Val2466Ala)	rs169547	Non-synonymous SNV	Benign	c.7397T>C (p.Val2466Ala)	rs169547	Non-synonymous SNV	Benign
				c.8878C>G (p.Gln2960Glu)	-	Non-synonymous SNV	VUS	c.8878C>G (p.Gln2960Glu)	-	Non-synonymous SNV	VUS
				c.8881_8884del (p.Gly2961fs)	-	Frameshift deletion	VUS	c.8881_8884del (p.Gly2961fs)	-	Frameshift deletion	VUS
				c.8885T>A	-	Stopgain	VUS	c.8885T>A	-	Stopgain	VUS
				PALB2							
				c.3351-53delT	rs35294437	Intronic	VUS	c.3351-53delT	rs35294437	Intronic	VUS
				c.3114-51T>A	rs249936	Intronic	Benign	c.3114-51T>A	rs249936	Intronic	Benign
				c.2586+58C>T	rs249954	Intronic	Benign	c.2586+58C>T	rs249954	Intronic	Benign
				c.1676A>G (p.Gln559Arg)	rs152451	Non-synonymous SNV	Benign	c.1676A>G (p.Gln559Arg)	rs152451	Non-synonymous SNV	Benign

				c.1675_1676insGAGTGAAAGGTAAATCAAGATGTGTGCTCTTCCGACTCC (p.Q559delinsRVKGKSRCVLFRLQ)	-	Non-frameshift insertion	VUS	c.1675_1676insGAGTGAAAGGTAAATCAAGATGTGTGCTCTTCCGACTCC (p.Q559delinsRVKGKSRCVLFRLQ)	-	Non-frameshift insertion	VUS
S4	ACT	53	PR	***BRCA1***							
				No mutations				c.4308T>C (p.Ser1436=)	rs1060915	Synonymous SNV	Benign
				BRCA2							
				c.7397T>C (p.Val2466Ala)	rs169547	Non-synonymous SNV	Benign	c.7397T>C (p.Val2466Ala)	rs169547	Non-synonymous SNV	Benign
				c.6513G>C (p.Val2171=)	rs206076	Synonymous SNV	Benign	c.6513G>C (p.Val2171=)	rs206076	Synonymous SNV	Benign
				c.8878C>T p.(Gln2960Ter)	-	Non-synonymous SNV	Pathogenic	c.8878C>T p.(Gln2960Ter)	-	Non-synonymous SNV	Pathogenic
				c.8881_8884del (p.Gly2961fs)	-	Frameshift deletion	VUS	c.8881_8884del (p.Gly2961fs)	-	Frameshift deletion	VUS
				c.8885T>A	-	Stopgain	VUS	c.8885T>A	-	Stopgain	VUS
				c.4563A>G (p.Leu1521=)	rs206075	Synonymous SNV	Benign	c.4563A>G (p.Leu1521=)	rs206075	Synonymous SNV	Benign
				c.3396A>G (p.Lys1132=)	rs1801406	Synonymous SNV	Little Clinical Significance	c.3396A>G (p.Lys1132=)	rs1801406	Synonymous SNV	Little Clinical Significance
				c.7242A>G (p.Ser2414=)	rs1799955	Synonymous SNV	Little Clinical Significance	Elimination			
				PALB2							
				c.3114-51T>A	rs249936	Intronic	Benign	c.3114-51T>A	rs249936	Intronic	Benign
				c.2586+58C>T	rs249954	Intronic	Benign	c.2586+58C>T	rs249954	Intronic	Benign
				c.1676A>G (p.Gln559Arg)	rs152451	Non-synonymous SNV	Benign	Elimination			
				c.1675_1676insGAGTGAAAGGTAAATCAAGATGTGTGCTCTTCCGACTCC (p.Q559delinsRVKGKSRCVLFRLQ)	-	Non-frameshift insertion	VUS	c.1675_1676insGAGTGAAAGGTAAATCAAGATGTGTGCTCTTCCGACTCC (p.Q559delinsRVKGKSRCVLFRLQ)	-	Non-frameshift insertion	VUS
				No mutations				c.3465dupT (p. Asp1156 Gln1157delinsX)	-	Stopgain	VUS
								c.3351-53delT	rs35294437	Intronic	VUS
								c.1706_1707del (p.Lys569fs)	rs1060502759	Frameshift deletion	Pathogenic
								c.1706delA (p.Lys569fs)	-	Frameshift deletion	VUS
								c.1676A>G (p.Gln559Arg)	rs152451	Non-synonymous SNV	Benign
S5	ACT	85	PR	***BRCA1***							
				c.4807_4821del (p.Pro1603_Val1607del)	rs80359888	Non-frameshift deletion	VUS	c.4807_4821del (p.Pro1603_Val1607del)	rs80359888	Non-frameshift deletion	VUS
				c.2609C>G (p.Ala870Gly)	rs1060502324	Non-synonymous SNV	VUS	c.2609C>G (p.Ala870Gly)	rs1060502324	Non-synonymous SNV	VUS
				c.2608G>A (p.Ala870Thr)	rs753256448	Non-synonymous SNV	VUS	c.2608G>A (p.Ala870Thr)	rs753256448	Non-synonymous SNV	VUS
				BRCA2							

				c.7397T>C (p.Val2466Ala)	rs169547	Non-synonymous SNV	Benign	c.7397T>C (p.Val2466Ala)	rs169547	Non-synonymous SNV	Benign
				c.6513G>C (p.Val2171=)	rs206076	Synonymous SNV	Benign	c.6513G>C (p.Val2171=)	rs206076	Synonymous SNV	Benign
				c.4563A>G (p.Leu1521=)	rs206075	Synonymous SNV	Benign	c.4563A>G (p.Leu1521=)	rs206075	Synonymous SNV	Benign
				c.3396A>G (p.Lys1132=)	rs1801406	Synonymous SNV	Little Clinical Significance	c.3396A>G (p.Lys1132=)	rs1801406	Synonymous SNV	Little Clinical Significance
				c.4288_4289insGGAACTGAGT (p.Thr1430_1431Ala_delinsRNX)	-	Stopgain	VUS	c.4288_4289insGGAACTGAGT (p.Thr1430_1431Ala_delinsRNX)	-	Stopgain	VUS
				c.7242A>G (p.Ser2414=)	rs1799955	Synonymous SNV	Little Clinical Significance	c.7242A>G (p.Ser2414=)	rs1799955	Synonymous SNV	Little Clinical Significance
				c.8878C>T p.(Gln2960Ter)	-	Non-synonymous SNV	Pathogenic	Elimination			
				c.8881_8884del (p.Gly2961fs)	-	Frameshift deletion	VUS				
				c.8885T>A	-	Stopgain	VUS				
				No mutations				c.9090delA (p.T3030fs)	rs397507420	Frameshift deletion	Pathogenic
								c.9090dupA (p.Thr3030fs)	-	Frameshift duplication	VUS
								c.7108_7109insCAT (p.K2370delinsTX)	-	Stopgain	VUS
								c.7110_7111insATATGTGGG (p.Lys2370delinsKICG)	-	Non-frameshift insertion	VUS
				PALB2							
				c.3114-51T>A	rs249936	Intronic	Benign	c.3114-51T>A	rs249936	Intronic	Benign
				c.2552delA (p.Asn851fs)	-	Frameshift deletion	VUS	Elimination			
				c.95_96insCGGAAG (p.Leu32 delinsLGR)	-	Non-frameshift insertion	VUS	Elimination			
				c.92_94del (p.31_32del)	-	Non-frameshift deletion	VUS	Elimination			
				c.94C>G (p.Leu32Val)	rs151316635	Non-synonymous SNV	Conflicting interpretations of pathogenicity	Elimination			
				No mutations				c.2586+58C>T	rs249954	Intronic	Benign
				No mutations				c.1693delA (p.Ser565fs)	-	Frameshift deletion	VUS
Kh2	AT	83	PR	***BRCA1***							
				c.2609C>G (p.Ala870Gly)	rs1060502324	Non-synonymous SNV	VUS	Elimination			
				c.2608G>A (p.Ala870Thr)	rs753256448	Non-synonymous SNV	VUS	Elimination			
				c.2612_2613insT (p.Phe872fs)	rs80357948	Frameshift insertion	Pathogenic	Elimination			
				c.1067A>G (p.Gln356Arg)	rs1799950	Non-synonymous SNV	VUS	c.1067A>G (p.Gln356Arg)	rs1799950	Non-synonymous SNV	VUS

				BRCA2							
				c.7397T>C (p.Val2466Ala)	rs169547	Non-synonymous SNV	Benign	c.7397T>C (p.Val2466Ala)	rs169547	Non-synonymous SNV	Benign
				c.6513G>C (p.Val2171=)	rs206076	Synonymous SNV	Benign	c.6513G>C (p.Val2171=)	rs206076	Synonymous SNV	Benign
				c.4563A>G (p.Leu1521=)	rs206075	Synonymous SNV	Benign	c.4563A>G (p.Leu1521=)	rs206075	Synonymous SNV	Benign
				c.1114A>C (p.Asn372His)	rs144848	Non-synonymous SNV	Little Clinical Significance	c.1114A>C (p.Asn372His)	rs144848	Non-synonymous SNV	Little Clinical Significance
				c.3807T>C (p.Val1269=)	rs543304	Synonymous SNV	Likely benign	c.3807T>C (p.Val1269=)	rs543304	Synonymous SNV	Likely benign
				c.4288_4289insGGAACTGAGT (p.Thr1430_1431Ala_delinsRNX)	-	Stopgain	VUS	Elimination			
				c.9090delA (p.T3030fs)	rs397507420	Frameshift deletion	Pathogenic	c.9090delA (p.T3030fs)	rs397507420	Frameshift deletion	Pathogenic
				c.9090dupA (p.Thr3030fs)	-	Frameshift duplication	VUS	c.9090dupA (p.Thr3030fs)	-	Frameshift duplication	VUS
				No mutations				c.3823_3824insAGCAGTTCC (p.Ile1275delinsKQFL)	-	Frameshift insertion	VUS
								c.3824_3825del p.(Ile1275ArgfsTer4)	-	Frameshift deletion	Pathogenic
				PALB2							
				c.3351-53delT	rs35294437	Intronic	VUS	c.3351-53delT	rs35294437	Intronic	VUS
				c.3114-51T>A	rs249936	Intronic	Benign	c.3114-51T>A	rs249936	Intronic	Benign
				c.2586+58C>T	rs249954	Intronic	Benign	c.2586+58C>T	rs249954	Intronic	Benign
				c.1676A>G (p.Gln559Arg)	rs152451	Non-synonymous SNV	Benign	c.1676A>G (p.Gln559Arg)	rs152451	Non-synonymous SNV	Benign
				c.1675_1676insGAGTGAAAGGTAAATCAAGATGTGTGCTCTTCCGACTCC (p.Q559delinsRVKGKSRCVLFRLQ)	-	Non-frameshift insertion	VUS	c.1675_1676insGAGTGAAAGGTAAATCAAGATGTGTGCTCTTCCGACTCC (p.Q559delinsRVKGKSRCVLFRLQ)	-	Non-frameshift insertion	VUS
				BRCA1							
				No mutations				No mutations			
				BRCA2							
				c.7397T>C (p.Val2466Ala)	rs169547	Non-synonymous SNV	Benign	c.7397T>C (p.Val2466Ala)	rs169547	Non-synonymous SNV	Benign
				c.6513G>C (p.Val2171=)	rs206076	Synonymous SNV	Benign	c.6513G>C (p.Val2171=)	rs206076	Synonymous SNV	Benign
				c.4563A>G (p.Leu1521=)	rs206075	Synonymous SNV	Benign	c.4563A>G (p.Leu1521=)	rs206075	Synonymous SNV	Benign
Sh2	Taxotere	80	PR	c.3396A>G (p.Lys1132=)	rs1801406	Synonymous SNV	Little Clinical Significance	c.3396A>G (p.Lys1132=)	rs1801406	Synonymous SNV	Little Clinical Significance
				c.7242A>G (p.Ser2414=)	rs1799955	Synonymous SNV	Little Clinical Significance	c.7242A>G (p.Ser2414=)	rs1799955	Synonymous SNV	Little Clinical Significance
				c.8878C>T p.(Gln2960Ter)	-	Non-synonymous SNV	Pathogenic	Elimination			

				c.8881_8884del (p.Gly2961fs)	-	Frameshift deletion	VUS				
				c.8885T>A	-	Stopgain	VUS				
				No mutations				c.3823_3824insAGCAGTTCC (p.Ile1275delinsKQFL)	-	Frameshift insertion	VUS
								c.3824_3825del p.(Ile1275ArgfsTer4)	-	Frameshift deletion	Pathogenic
								c.9090delA (p.T3030fs)	rs397507420	Frameshift deletion	Pathogenic
								c.9090dupA (p.Thr3030fs)	-	Frameshift duplication	VUS
				PALB2							
				c.3114-51T>A	rs249936	Intronic	Benign	c.3114-51T>A	rs249936	Intronic	Benign
				No mutations				c.3351-53delT	rs35294437	Intronic	VUS
				No mutations				c.1686delG (p.Gly562fs)	-	Frameshift deletion	Pathogenic
B5	Taxotere	100	CR	***BRCA1***							
				No mutations				No mutations			
				BRCA2							
				c.7397T>C (p.Val2466Ala)	rs169547	Non-synonymous SNV	Benign	Elimination			
				c.6513G>C (p.Val2171=)	rs206076	Synonymous SNV	Benign				
				c.8878C>T p.(Gln2960Ter)	-	Non-synonymous SNV	Pathogenic				
				c.8881_8884del (p.Gly2961fs)	-	Frameshift deletion	VUS				
				c.8885T>A	-	Stopgain	VUS				
				c.4563A>G (p.Leu1521=)	rs206075	Synonymous SNV	Benign				
				c.3807T>C (p.Val1269=)	rs543304	Synonymous SNV	Likely benign				
				PALB2							
				c.3114-51T>A	rs249936	Intronic	Benign	Elimination			
				c.2586+58C>T	rs249954	Intronic	Benign				
				c.1676A>G (p.Gln559Arg)	rs152451	Non-synonymous SNV	Benign				
A2	Taxotere	25	ST	***BRCA1***							
				No mutations				No mutations			
				BRCA2							
				No mutations				c.3396A>G (p.Lys1132=)	rs1801406	Synonymous SNV	Little Clinical Significance
				c.4563A>G (p.Leu1521=)	rs206075	Synonymous SNV	Benign	c.4563A>G (p.Leu1521=)	rs206075	Synonymous SNV	Benign
				c.6513G>C (p.Val2171=)	rs206076	Synonymous SNV	Benign	c.6513G>C (p.Val2171=)	rs206076	Synonymous SNV	Benign
				No mutations				c.7242A>G (p.Ser2414=)	rs1799955	Synonymous SNV	Little Clinical Significance
				c.1514T>C (p.Ile505Thr)	rs28897708	Non-synonymous SNV	Benign	c.1514T>C (p.Ile505Thr)	rs28897708	Non-synonymous SNV	Benign

				c.7397T>C (p.Val2466Ala)	rs169547	Non-synonymous SNV	Benign	c.7397T>C (p.Val2466Ala)	rs169547	Non-synonymous SNV	Benign
				PALB2							
				c.3114-51T>A	rs249936	Intronic	Benign	c.3114-51T>A	rs249936	Intronic	Benign
				c.2586+58C>T	rs249954	Intronic	Benign	c.2586+58C>T	rs249954	Intronic	Benign
B6	Taxotere	61	PR	***BRCA1***							
				No mutations				No mutations			
				BRCA2							
				c.3396A>G (p.Lys1132=)	rs1801406	Synonymous SNV	Little Clinical Significance	Elimination			
				c.4563A>G (p.Leu1521=)	rs206075	Synonymous SNV	Benign				
				c.6513G>C (p.Val2171=)	rs206076	Synonymous SNV	Benign				
				c.7242A>G (p.Ser2414=)	rs1799955	Synonymous SNV	Little Clinical Significance				
				c.7397T>C (p.Val2466Ala)	rs169547	Non-synonymous SNV	Benign				
				c.8208_8209insAG (p.Leu2737fs)	rs483353122	Frameshift insertion	Pathogenic				
				PALB2							
				c.3114-51T>A	rs249936	Intronic	Benign	c.3114-51T>A	rs249936	Intronic	Benign
K2	ACT	100	CR	***BRCA1***							
				c.4900A>G (p.Ser1634Gly)	rs1799966	Non-synonymous SNV	Benign	No date			
				c.2311T>C (p.Leu771=)	rs16940	Synonymous SNV	Benign				
				c.2612C>T (p.Pro871Leu)	rs799917	Non-synonymous SNV	Benign				
				c.2470_2471insTTCCGATCTTAGTCC (p.Pro 824delinsLPILVP)	-	Non-frameshift insertion	VUS				
				c.3548A>G (p.Lys1183Arg)	rs16942	Non-synonymous SNV	Benign				
				c.4308T>C (p.Ser1436=)	rs1060915	Synonymous SNV	Benign				
				c.3113A>G (p.Glu1038Gly)	rs16941	Non-synonymous SNV	Benign				
				c.2082C>T (p.Ser694=)	rs1799949	Synonymous SNV	Benign				
				BRCA2							
				c.2229T>C (p.His743=)	rs1801499	Synonymous SNV	Benign	No date			
				c.3396A>G (p.Lys1132=)	rs1801406	Synonymous SNV	Little Clinical Significance				
				c.3823_3824insAGCAGTTCC (p.Ile1275delinsKQFL)	-	Frameshift insertion	VUS				
				c.4563A>G (p.Leu1521=)	rs206075	Synonymous SNV	Benign				
				c.6513G>C (p.Val2171=)	rs206076	Synonymous SNV	Benign				
				c.7242A>G (p.Ser2414=)	rs1799955	Synonymous SNV	Little Clinical Signific				

							ance	
				c.7397T>C (p.Val2466Ala)	rs169547	Non-synonymous SNV	Benign	
				c.8208_8209insAG (p.Leu2737fs)	rs483353122	Frameshift insertion	Pathogenic	
				c.9090delA (p.T3030fs)	rs397507420	Frameshift deletion	Pathogenic	
				c.9090dupA (p.Thr3030fs)	-	Frameshift duplication	VUS	
				c.3824_3825del p.(Ile1275ArgfsTer4)	-	Frameshift deletion	Pathogenic	
				PALB2				
				c.3114-51T>A	rs249936	Intronic	Benign	No date
Z1	Taxotere	50	PR	***BRCA1***				
				c.4308T>C (p.Ser1436=)	rs1060915	Synonymous SNV	Benign	No date
				c.2311T>C (p.Leu771=)	rs16940	Synonymous SNV	Benign	
				c.2082C>T (p.Ser694=)	rs1799949	Synonymous SNV	Benign	
				c.4900A>G (p.Ser1634Gly)	rs1799966	Non-synonymous SNV	Benign	
				c.3548A>G (p.Lys1183Arg)	rs16942	Non-synonymous SNV	Benign	
				c.3113A>G (p.Glu1038Gly)	rs16941	Non-synonymous SNV	Benign	
				c.2612C>T (p.Pro871Leu)	rs799917	Non-synonymous SNV	Benign	
				BRCA2				
				c.4563A>G (p.Leu1521=)	rs206075	Synonymous SNV	Benign	No date
				c.6513G>C (p.Val2171=)	rs206076	Synonymous SNV	Benign	
				c.7397T>C (p.Val2466Ala)	rs169547	Non-synonymous SNV	Benign	
				PALB2				
				c.3114-51T>A	rs249936	Intronic	Benign	No date
K3	Taxotere	-121	P	***BRCA1***				
				c.4308T>C (p.Ser1436=)	rs1060915	Synonymous SNV	Benign	No date
				c.2311T>C (p.Leu771=)	rs16940	Synonymous SNV	Benign	
				c.2082C>T (p.Ser694=)	rs1799949	Synonymous SNV	Benign	
				c.4900A>G (p.Ser1634Gly)	rs1799966	Non-synonymous SNV	Benign	
				c.3548A>G (p.Lys1183Arg)	rs16942	Non-synonymous SNV	Benign	
				c.3113A>G (p.Glu1038Gly)	rs16941	Non-synonymous SNV	Benign	
				c.2612C>T (p.Pro871Leu)	rs799917	Non-synonymous SNV	Benign	
				c.2077G>A (p.Asp693Asn)	rs4986850	Non-synonymous SNV	Benign	
				BRCA2				
				c.3396A>G (p.Lys1132=)	rs1801406	Synonymous SNV	Little Clinical Significance	No date
				c.4563A>G (p.Leu1521=)	rs206075	Synonymous SNV	Benign	
				c.6513G>C (p.Val2171=)	rs206076	Synonymous SNV	Benign	
				c.7397T>C (p.Val2466Ala)	rs169547	Non-synonymous SNV	Benign	
				c.-26G>A	rs1799943	5 prime UTR variant	VUS	
				PALB2				
				c.3114-51T>A	rs249936	Intronic	Benign	No date

				c.1010T>C (p.Leu337Ser)	rs45494092	Non-synonymous SNV	Benign	
L3	Taxotere	-149	P	***BRCA1***				
				c.4308T>C (p.Ser1436=)	rs1060915	Synonymous SNV	Benign	No date
				c.2311T>C (p.Leu771=)	rs16940	Synonymous SNV	Benign	
				c.2082C>T (p.Ser694=)	rs1799949	Synonymous SNV	Benign	
				c.4900A>G (p.Ser1634Gly)	rs1799966	Non-synonymous SNV	Benign	
				c.3548A>G (p.Lys1183Arg)	rs16942	Non-synonymous SNV	Benign	
				c.3113A>G (p.Glu1038Gly)	rs16941	Non-synonymous SNV	Benign	
				c.2612C>T (p.Pro871Leu)	rs799917	Non-synonymous SNV	Benign	
				c.4035delA (p.Glu1345fs)	rs80357711	Frameshift deletion	Pathogenic	
				BRCA2				
				c.4563A>G (p.Leu1521=)	rs206075	Synonymous SNV	Benign	No date
				c.6513G>C (p.Val2171=)	rs206076	Synonymous SNV	Benign	
				c.1114A>C (p.Asn372His)	rs144848	Non-synonymous SNV	Little Clinical Significance	
				c.7397T>C (p.Val2466Ala)	rs169547	Non-synonymous SNV	Benign	
				PALB2				
				c.3351-53delT	rs35294437	Intronic	VUS	No data
				c.3114-51T>A	rs249936	Intronic	Benign	
				c.2586+58C>T	rs249954	Intronic	Benign	
				c.1676A>G (p.Gln559Arg)	rs152451	Non-synonymous SNV	Benign	
N2	Taxotere	84	PR	***BRCA1***				
				c.1067A>G (p.Gln356Arg)	rs1799950	Non-synonymous SNV	VUS	No data
				BRCA2				
				c.4563A>G (p.Leu1521=)	rs206075	Synonymous SNV	Benign	No data
				c.6513G>C (p.Val2171=)	rs206076	Synonymous SNV	Benign	
				c.7397T>C (p.Val2466Ala)	rs169547	Non-synonymous SNV	Benign	
				PALB2				
				c.3114-51T>A	rs249936	Intronic	Benign	No data
N3	Taxotere	60	PR	***BRCA1***				
				c.5019G>A (p.Met1652Ile)	rs1799967	Non-synonymous SNV	Benign	No data
				c.4900A>G (p.Ser1634Gly)	rs1799966	Non-synonymous SNV	Benign	
				c.4308T>C (p.Ser1436=)	rs1060915	Synonymous SNV	Benign	
				c.3548A>G (p.Lys1183Arg)	rs16942	Non-synonymous SNV	Benign	
				c.3113A>G (p.Glu1038Gly)	rs16941	Non-synonymous SNV	Benign	
				c.2612C>T (p.Pro871Leu)	rs799917	Non-synonymous SNV	Benign	
				c.2311T>C (p.Leu771=)	rs16940	Synonymous SNV	Benign	
				c.2082C>T (p.Ser694=)	rs1799949	Synonymous SNV	Benign	
				BRCA2				
				c.-26G>A	rs1799943	5 prime UTR variant	VUS	No data
				c.1114A>C (p.Asn372His)	rs144848	Non-synonymous SNV	Little	

							Clinical Significance	
				c.3396A>G (p.Lys1132=)	rs1801406	Synonymous SNV	Little Clinical Significance	
				c.4563A>G (p.Leu1521=)	rs206075	Synonymous SNV	Benign	
				c.6513G>C (p.Val2171=)	rs206076	Synonymous SNV	Benign	
				c.7242A>G (p.Ser2414=)	rs1799955	Synonymous SNV	Little Clinical Significance	
				c.7397T>C (p.Val2466Ala)	rs169547	Non-synonymous SNV	Benign	
				PALB2				
				c.3114-51T>A	rs249936	Intronic	Benign	No data
P2	Taxotere	66	PR	***BRCA1***				
				c.4900A>G (p.Ser1634Gly)	rs1799966	Non-synonymous SNV	Benign	No data
				c.4308T>C (p.Ser1436=)	rs1060915	Synonymous SNV	Benign	
				c.3548A>G (p.Lys1183Arg)	rs16942	Non-synonymous SNV	Benign	
				c.3113A>G (p.Glu1038Gly)	rs16941	Non-synonymous SNV	Benign	
				c.2612C>T (p.Pro871Leu)	rs799917	Non-synonymous SNV	Benign	
				c.2311T>C (p.Leu771=)	rs16940	Synonymous SNV	Benign	
				c.2082C>T (p.Ser694=)	rs1799949	Synonymous SNV	Benign	
				c.1067A>G (p.Gln356Arg)	rs1799950	Non-synonymous SNV	VUS	
				BRCA2				
				c.4563A>G (p.Leu1521=)	rs206075	Synonymous SNV	Benign	No data
				c.6513G>C (p.Val2171=)	rs206076	Synonymous SNV	Benign	
				c.7397T>C (p.Val2466Ala)	rs169547	Non-synonymous SNV	Benign	
				PALB2				
				c.3351-53delT	rs35294437	Intronic	VUS	No data
				c.3114-51T>A	rs249936	Intronic	Benign	
				c.2586+58C>T	rs249954	Intronic	Benign	
				c.1676A>G (p.Gln559Arg)	rs152451	Non-synonymous SNV	Benign	
R3	Taxotere	61	PR	***BRCA1***				
				No mutations				No data
				BRCA2				
				c.-26G>A	rs1799943	5 prime UTR variant	VUS	No data
				c.3396A>G (p.Lys1132=)	rs1801406	Synonymous SNV	Little Clinical Significance	
				c.4563A>G (p.Leu1521=)	rs206075	Synonymous SNV	Benign	
				c.6513G>C (p.Val2171=)	rs206076	Synonymous SNV	Benign	
				c.7242A>G (p.Ser2414=)	rs1799955	Synonymous SNV	Little	

							Clinical Significance	
				c.7397T>C (p.Val2466Ala)	rs169547	Non-synonymous SNV	Benign	
				PALB2				
				c.3114-51T>A	rs249936	Intronic	Benign	No data
				c.2586+58A>G	rs249954	Intronic	Benign	
S6	Taxotere	80	PR	***BRCA1***				
				c.4900A>G (p.Ser1634Gly)	rs1799966	Non-synonymous SNV	Benign	No data
				c.4308T>C (p.Ser1436=)	rs1060915	Synonymous SNV	Benign	
				c.3548A>G (p.Lys1183Arg)	rs16942	Non-synonymous SNV	Benign	
				c.3113A>G (p.Glu1038Gly)	rs16941	Non-synonymous SNV	Benign	
				c.2612C>T (p.Pro871Leu)	rs799917	Non-synonymous SNV	Benign	
				c.2311T>C (p.Leu771=)	rs16940	Synonymous SNV	Benign	
				c.2082C>T (p.Ser694=)	rs1799949	Synonymous SNV	Benign	
				c.2077G>A (p.Asp693Asn)	rs4986850	Non-synonymous SNV	Benign	
				BRCA2				
				c.-26G>A	rs1799943	5 prime UTR variant	VUS	No data
				c.3396A>G (p.Lys1132=)	rs1801406	Synonymous SNV	Little Clinical Significance	
				c.4563A>G (p.Leu1521=)	rs206075	Synonymous SNV	Benign	
				c.6513G>C (p.Val2171=)	rs206076	Synonymous SNV	Benign	
				c.7242A>G (p.Ser2414=)	rs1799955	Synonymous SNV	Little Clinical Significance	
				c.7397T>C (p.Val2466Ala)	rs169547	Non-synonymous SNV	Benign	
				PALB2				
				c.3114-51T>A	rs249936	Intronic	Benign	No data
F1	Taxotere	17	ST	***BRCA1***				
				c.1067A>G (p.Gln356Arg)	rs1799950	Non-synonymous SNV	VUS	No data
				BRCA2				
				c.-26G>A	rs1799943	5 prime UTR variant	VUS	No data
				c.3396A>G (p.Lys1132=)	rs1801406	Synonymous SNV	Little Clinical Significance	
				c.4563A>G (p.Leu1521=)	rs206075	Synonymous SNV	Benign	
				c.6513G>C (p.Val2171=)	rs206076	Synonymous SNV	Benign	
				c.7242A>G (p.Ser2414=)	rs1799955	Synonymous SNV	Little Clinical Significance	

				c.7397T>C (p.Val2466Ala)	rs169547	Non-synonymous SNV	Benign	
				PALB2				
				c.3114-51T>A	rs249936	Intronic	Benign	No data
				c.2586+58C>T	rs249954	Intronic	Benign	
F2	Taxotere	92	PR	***BRCA1***				
				c.5329dupC (p.Gln1777fs)	rs397507247,rs397507246,rs431825413;rs80357906	Frameshift duplication	Pathogenic	No data
				BRCA2				
				c.1114A>C (p.Asn372His)	rs144848	Non-synonymous SNV	Little Clinical Significance	No data
				c.4563A>G (p.Leu1521=)	rs206075	Synonymous SNV	Benign	
				c.6513G>C (p.Val2171=)	rs206076	Synonymous SNV	Benign	
				c.7397T>C (p.Val2466Ala)	rs169547	Non-synonymous SNV	Benign	
				PALB2				
				c.3114-51T>A	rs249936	Intronic	Benign	No data
Sh3	Taxotere	20	ST	***BRCA1***				
				c.2612C>T (p.Pro871Leu)	rs799917	Non-synonymous SNV	Benign	No data
				BRCA2				
				c.-26G>A	rs1799943	5 prime UTR variant	VUS	No data
				c.865A>C (p.Asn289His)	rs766173	Non-synonymous SNV	Benign	
				c.1365A>G (p.Ser455=)	rs1801439	Synonymous SNV	Benign	
				c.2229T>C (p.His743=)	rs1801499	Synonymous SNV	Benign	
				c.2971A>G (p.Asn991Asp)	rs1799944	Non-synonymous SNV	Benign	
				c.3396A>G (p.Lys1132=)	rs1801406	Synonymous SNV	Little Clinical Significance	
				c.4258G>T (p.Asp1420Tyr)	rs28897727	Non-synonymous SNV	Benign	
				c.4563A>G (p.Leu1521=)	rs206075	Synonymous SNV	Benign	
				c.6513G>C (p.Val2171=)	rs206076	Synonymous SNV	Benign	
				c.7397T>C (p.Val2466Ala)	rs169547	Non-synonymous SNV	Benign	
				PALB2				
				c.3114-51T>A	rs249936	Intronic	Benign	No data
				c.2586+58C>T	rs249954	Intronic	Benign	
Y1	Taxotere	46	ST	***BRCA1***				
				c.4900A>G (p.Ser1634Gly)	rs1799966	Non-synonymous SNV	Benign	No data
				c.4308T>C (p.Ser1436=)	rs1060915	Synonymous SNV	Benign	
				c.3548A>G (p.Lys1183Arg)	rs16942	Non-synonymous SNV	Benign	
				c.3113A>G (p.Glu1038Gly)	rs16941	Non-synonymous SNV	Benign	
				c.2612C>T (p.Pro871Leu)	rs799917	Non-synonymous SNV	Benign	
				c.2311T>C (p.Leu771=)	rs16940	Synonymous SNV	Benign	

				c.2082C>T (p.Ser694=)	rs1799949	Synonymous SNV	Benign				
				BRCA2							
				c.865A>C (p.Asn289His)	rs766173	Non-synonymous SNV	Benign				
				c.1365A>G (p.Ser455=)	rs1801439	Synonymous SNV	Benign				
				c.2229T>C (p.His743=)	rs1801499	Synonymous SNV	Benign				
				c.2971A>G (p.Asn991Asp)	rs1799944	Non-synonymous SNV	Benign	No data			
				c.4563A>G (p.Leu1521=)	rs206075	Synonymous SNV	Benign				
				c.6513G>C (p.Val2171=)	rs206076	Synonymous SNV	Benign				
				c.7397T>C (p.Val2466Ala)	rs169547	Non-synonymous SNV	Benign				
				PALB2							
				c.3114-51T>A	rs249936	Intronic	Benign	No data			
				c.2586+58C>T	rs249954	Intronic	Benign				
K4	CP	40	ST	***BRCA1***							
				c.4900A>G (p.Ser1634Gly)	rs1799966	Non-synonymous SNV	Benign	c.4900A>G (p.Ser1634Gly)	rs1799966	Non-synonymous SNV	Benign
				c.4807_4821del (p.Pro1603_Val1607del)	rs80359888	Non-frameshift deletion	VUS	c.4807_4821del (p.Pro1603_Val1607del)	rs80359888	Non-frameshift deletion	VUS
				c.4308T>C (p.Ser1436=)	rs1060915	Synonymous SNV	Benign	c.4308T>C (p.Ser1436=)	rs1060915	Synonymous SNV	Benign
				c.3548A>G (p.Lys1183Arg)	rs16942	Non-synonymous SNV	Benign	c.3548A>G (p.Lys1183Arg)	rs16942	Non-synonymous SNV	Benign
				c.3113A>G (p.Glu1038Gly)	rs16941	Non-synonymous SNV	Benign	c.3113A>G (p.Glu1038Gly)	rs16941	Non-synonymous SNV	Benign
				c.2612C>T (p.Pro871Leu)	rs799917	Non-synonymous SNV	Benign	c.2612C>T (p.Pro871Leu)	rs799917	Non-synonymous SNV	Benign
				c.2470_2471insTTCCGATC TTAGTCC (p.Pro 824delinsLPILVP)	-	Non-frameshift insertion	VUS	c.2470_2471insTTCCGATC TTAGTCC (p.Pro 824delinsLPILVP)	-	Non-frameshift insertion	VUS
				c.2311T>C (p.Leu771=)	rs16940	Synonymous SNV	Benign	c.2311T>C (p.Leu771=)	rs16940	Synonymous SNV	Benign
				c.2082C>T (p.Ser694=)	rs1799949	Synonymous SNV	Benign	c.2082C>T (p.Ser694=)	rs1799949	Synonymous SNV	Benign
				c.1067A>G (p.Gln356Arg)	rs1799950	Non-synonymous SNV	VUS	c.1067A>G (p.Gln356Arg)	rs1799950	Non-synonymous SNV	VUS
				BRCA2							
				c.4563A>G (p.Leu1521=)	rs206075	Synonymous SNV	Benign	c.4563A>G (p.Leu1521=)	rs206075	Synonymous SNV	Benign
				c.6513G>C (p.Val2171=)	rs206076	Synonymous SNV	Benign	c.6513G>C (p.Val2171=)	rs206076	Synonymous SNV	Benign
				c.7397T>C (p.Val2466Ala)	rs169547	Non-synonymous SNV	Benign	c.7397T>C (p.Val2466Ala)	rs169547	Non-synonymous SNV	Benign
								c.8878C>T p.(Gln2960Ter)	-	Non-synonymous SNV	Pathogenic
								c.8881_8884del (p.Gly2961fs)	-	Frameshift deletion	VUS
				No mutations				c.8885T>A	-	Stopgain	VUS
								c.3823_3824insAGCAGTTC C (p.Ile1275delinsKQFL)	-	Frameshift insertion	VUS
								c.3824_3825del p.(Ile1275ArgfsTer4)	-	Frameshift deletion	Pathogenic
				c.4288_4289insGGAACTGA GT (p.Thr1430_1431Ala_delinsR NX)	-	Stopgain	VUS	Elimination			
				PALB2							
				c.3114-51T>A	rs249936	Intronic	Benign	c.3114-51T>A	rs249936	Intronic	Benign

				c.3351-53delT	rs35294437	Intronic	VUS	Elimination			
				No mutations				c.255dupA (p.Thr86fs)	-	Frameshift duplication	VUS
				No mutations				c.256delA (p.Thr86fs)	-	Frameshift deletion	VUS
K5	CP	75	PR	***BRCA1***							
				c.4900A>G (p.Ser1634Gly)	rs1799966	Non-synonymous SNV	Benign	c.4900A>G (p.Ser1634Gly)	rs1799966	Non-synonymous SNV	Benign
				c.2311T>C (p.Leu771=)	rs16940	Synonymous SNV	Benign	c.2311T>C (p.Leu771=)	rs16940	Synonymous SNV	Benign
				c.2612C>T (p.Pro871Leu)	rs799917	Non-synonymous SNV	Benign	c.2612C>T (p.Pro871Leu)	rs799917	Non-synonymous SNV	Benign
				c.3548A>G (p.Lys1183Arg)	rs16942	Non-synonymous SNV	Benign	c.3548A>G (p.Lys1183Arg)	rs16942	Non-synonymous SNV	Benign
				c.3113A>G (p.Glu1038Gly)	rs16941	Non-synonymous SNV	Benign	c.3113A>G (p.Glu1038Gly)	rs16941	Non-synonymous SNV	Benign
				c.2082C>T (p.Ser694=)	rs1799949	Synonymous SNV	Benign	c.2082C>T (p.Ser694=)	rs1799949	Synonymous SNV	Benign
				c.4308T>C (p.Ser1436=)	rs1060915	Synonymous SNV	Benign	c.4308T>C (p.Ser1436=)	rs1060915	Synonymous SNV	Benign
				c.5019G>A (p.Met1652Ile)	rs1799967	Non-synonymous SNV	Benign	Elimination			
				No mutations				c.2470_2471insTTCCGATC TTAGTCC (p. Pro 824delinsLPILVP)	-	Non-frameshift insertion	VUS
				BRCA2							
				c.4563A>G (p.Leu1521=)	rs206075	Synonymous SNV	Benign	c.4563A>G (p.Leu1521=)	rs206075	Synonymous SNV	Benign
				c.6513G>C (p.Val2171=)	rs206076	Synonymous SNV	Benign	c.6513G>C (p.Val2171=)	rs206076	Synonymous SNV	Benign
				c.7397T>C (p.Val2466Ala)	rs169547	Non-synonymous SNV	Benign	c.7397T>C (p.Val2466Ala)	rs169547	Non-synonymous SNV	Benign
				c.865A>C (p.Asn289His)	rs766173	Non-synonymous SNV	Benign	Elimination			
				c.1365A>G (p.Ser455=)	rs1801439	Synonymous SNV	Benign				
				c.2229T>C (p.His743=)	rs1801499	Synonymous SNV	Benign				
				c.2971A>G (p.Asn991Asp)	rs1799944	Non-synonymous SNV	Benign				
				c.7544C>T (p.Thr2515Ile)	rs28897744	Non-synonymous SNV	Benign				
				No mutations				c.8878C>T p.(Gln2960Ter)	-	Non-synonymous SNV	Pathoge nic
								c.8881_8884del (p.Gly2961fs)	-	Frameshift deletion	VUS
								c.8885T>A	-	Stopgain	VUS
				PALB2							
				c.3114-51T>A	rs249936	Intronic	Benign	c.3114-51T>A	rs249936	Intronic	Benign
V3	CP	100	CR	***BRCA1***							
				c.4900A>G (p.Ser1634Gly)	rs1799966	Non-synonymous SNV	Benign	No date			
				c.4308T>C (p.Ser1436=)	rs1060915	Synonymous SNV	Benign				
				c.3548A>G (p.Lys1183Arg)	rs16942	Non-synonymous SNV	Benign				
				c.3113A>G (p.Glu1038Gly)	rs16941	Non-synonymous SNV	Benign				
				c.2612C>T (p.Pro871Leu)	rs799917	Non-synonymous SNV	Benign				
				c.2470_2471insTTCCGATC TTAGTCC (p.Pro 824delinsLPILVP)	-	Non-frameshift insertion	VUS				
				c.2311T>C (p.Leu771=)	rs16940	Synonymous SNV	Benign				
				c.2082C>T (p.Ser694=)	rs1799949	Synonymous SNV	Benign				
				BRCA2							
				c.1114A>C (p.Asn372His)	rs144848	Non-synonymous SNV	Little Clinical	No date			

							Significance				
				c.3396A>G (p.Lys1132=)	rs1801406	Synonymous SNV	Little Clinical Significance				
				c.3823_3824insAGCAGTTCC (p.Ile1275delinsKQFL)	-	Frameshift insertion	VUS				
				c.3824_3825del p.(Ile1275ArgfsTer4)	-	Frameshift deletion	Pathogenic				
				c.4563A>G (p.Leu1521=)	rs206075	Synonymous SNV	Benign				
				c.6513G>C (p.Val2171=)	rs206076	Synonymous SNV	Benign				
				c.7397T>C (p.Val2466Ala)	rs169547	Non-synonymous SNV	Benign				
				PALB2							
				c.3114-51T>A	rs249936	Intronic	Benign	No date			
				c.747T>A (p.Pro249=)	-	Synonymous SNV	VUS				
P3	CP	55	PR	***BRCA1***							
				c.2609C>G (p.Ala870Gly)	rs1060502324	Non-synonymous SNV	VUS	No date			
				c.2608G>A (p.Ala870Thr)	rs753256448	Non-synonymous SNV	VUS				
				BRCA2							
				c.1114A>C (p.Asn372His)	rs144848	Non-synonymous SNV	Little Clinical Significance	No date			
				c.4288_4289insGGAACTGAGT (p.Thr1430_1431Ala_delinsRNX)	-	Stopgain	VUS				
				c.4563A>G (p.Leu1521=)	rs206075	Synonymous SNV	Benign				
				c.6513G>C (p.Val2171=)	rs206076	Synonymous SNV	Benign				
				c.7397T>C (p.Val2466Ala)	rs169547	Non-synonymous SNV	Benign				
				c.9090delA (p.T3030fs)	rs397507420	Frameshift deletion	Pathogenic				
				c.9090dupA (p.Thr3030fs)	-	Frameshift duplication	VUS				
				PALB2							
				c.3351-53delT	rs35294437	Intronic	VUS	No date			
				c.3114-51T>A	rs249936	Intronic	Benign				
P4	CP	40	ST	***BRCA1***							
				c.4807_4821del (p.Pro1603_Val1607del)	rs80359888	Non-frameshift deletion	VUS	Elimination			
				c.1067A>G (p.Gln356Arg)	rs1799950	Non-synonymous SNV	VUS				
				No mutations				c.2609C>G (p.Ala870Gly)	rs1060502324	Non-synonymous SNV	VUS
								c.2608G>A (p.Ala870Thr)	rs753256448	Non-synonymous SNV	VUS
				BRCA2							
				c.1114A>C (p.Asn372His)	rs144848	Non-synonymous SNV	Little Clinical	c.1114A>C (p.Asn372His)	rs144848	Non-synonymous SNV	Little Clinical

							Significance				Significance
				c.3396A>G (p.Lys1132=)	rs1801406	Synonymous SNV	Little Clinical Significance	c.3396A>G (p.Lys1132=)	rs1801406	Synonymous SNV	Little Clinical Significance
				c.4288_4289insGGAACTGAGT (p.Thr1430_1431Ala_delinsRNX)	-	Stopgain	VUS	c.4288_4289insGGAACTGAGT (p.Thr1430_1431Ala_delinsRNX)	-	Stopgain	VUS
				c.4563A>G (p.Leu1521=)	rs206075	Synonymous SNV	Benign	c.4563A>G (p.Leu1521=)	rs206075	Synonymous SNV	Benign
				c.6513G>C (p.Val2171=)	rs206076	Synonymous SNV	Benign	c.6513G>C (p.Val2171=)	rs206076	Synonymous SNV	Benign
				c.7397T>C (p.Val2466Ala)	rs169547	Non-synonymous SNV	Benign	c.7397T>C (p.Val2466Ala)	rs169547	Non-synonymous SNV	Benign
				c.7108_7109insCAT (p.K2370delinsTX)	-	Stopgain	VUS	Elimination			
				c.7110_7111insATATGTGGG (p.Lys2370delinsKICG)	-	Non-frameshift insertion	VUS				
				c.8878C>T p.(Gln2960Ter)	-	Non-synonymous SNV	Pathogenic				
				c.8881_8884del (p.Gly2961fs)	-	Frameshift deletion	VUS				
				c.8885T>A	-	Stopgain	VUS				
				No mutations				c.7242A>G (p.Ser2414=)	rs1799955	Synonymous SNV	Little Clinical Significance
								c.9090delA (p.T3030fs)	rs397507420	Frameshift deletion	Pathogenic
								c.9090dupA (p.Thr3030fs)	-	Frameshift duplication	VUS
				PALB2							
				c.3351-53delT	rs35294437	Intronic	VUS	Elimination			
				No mutations				c.2552delA (p.Asn851fs)	-	Frameshift deletion	VUS
P5	CP	12	ST	***BRCA1***							
				c.4900A>G (p.Ser1634Gly)	rs1799966	Non-synonymous SNV	Benign	c.4900A>G (p.Ser1634Gly)	rs1799966	Non-synonymous SNV	Benign
				c.4308T>C (p.Ser1436=)	rs1060915	Synonymous SNV	Benign	c.4308T>C (p.Ser1436=)	rs1060915	Synonymous SNV	Benign
				c.3548A>G (p.Lys1183Arg)	rs16942	Non-synonymous SNV	Benign	c.3548A>G (p.Lys1183Arg)	rs16942	Non-synonymous SNV	Benign
				c.3113A>G (p.Glu1038Gly)	rs16941	Non-synonymous SNV	Benign	c.3113A>G (p.Glu1038Gly)	rs16941	Non-synonymous SNV	Benign
				c.2612C>T (p.Pro871Leu)	rs799917	Non-synonymous SNV	Benign	c.2612C>T (p.Pro871Leu)	rs799917	Non-synonymous SNV	Benign
				c.2470_2471insTTCCGATCTTAGTCC (p.Pro 824delinsLPILVP)	-	Non-frameshift insertion	VUS	c.2470_2471insTTCCGATCTTAGTCC (p.Pro 824delinsLPILVP)	-	Non-frameshift insertion	VUS
				c.2311T>C (p.Leu771=)	rs16940	Synonymous SNV	Benign	c.2311T>C (p.Leu771=)	rs16940	Synonymous SNV	Benign
				c.2082C>T (p.Ser694=)	rs1799949	Synonymous SNV	Benign	c.2082C>T (p.Ser694=)	rs1799949	Synonymous SNV	Benign
				No mutations				c.4900A>G (p.Ser1634Gly)	rs1799966	Non-synonymous SNV	Benign
								c.4807_4821del (p.Pro1603_Val1607del)	rs80359888	Non-frameshift deletion	VUS

				BRCA2							
				c.3807T>C (p.Val1269=)	rs543304	Synonymous SNV	Likely benign	c.3807T>C (p.Val1269=)	rs543304	Synonymous SNV	Likely benign
				c.4563A>G (p.Leu1521=)	rs206075	Synonymous SNV	Benign	c.4563A>G (p.Leu1521=)	rs206075	Synonymous SNV	Benign
				c.6513G>C (p.Val2171=)	rs206076	Synonymous SNV	Benign	c.6513G>C (p.Val2171=)	rs206076	Synonymous SNV	Benign
				c.7397T>C (p.Val2466Ala)	rs169547	Non-synonymous SNV	Benign	c.7397T>C (p.Val2466Ala)	rs169547	Non-synonymous SNV	Benign
				c.8878C>T p.(Gln2960Ter)	-	Non-synonymous SNV	Pathogenic	Elimination			
				c.8881_8884del (p.Gly2961fs)	-	Frameshift deletion	VUS				
				c.8885T>A	-	Stopgain	VUS				
				No mutations				c.7108_7109insCAT (p.K2370delinsTX)	-	Stopgain	VUS
								c.7110_7111insATATGTGGG (p.Lys2370delinsKICG)	-	Non-frameshift insertion	VUS
								c.9090delA (p.T3030fs)	rs397507420	Frameshift deletion	Pathogenic
								c.9090dupA (p.Thr3030fs)	-	Frameshift duplication	VUS
				PALB2							
				c.3114-51T>A	rs249936	Intronic	Benign	c.3114-51T>A	rs249936	Intronic	Benign
				c.3351-53delT	rs35294437	Intronic	VUS	Elimination			
				BRCA1							
				c.4900A>G (p.Ser1634Gly)	rs1799966	Non-synonymous SNV	Benign	c.4900A>G (p.Ser1634Gly)	rs1799966	Non-synonymous SNV	Benign
				c.4308T>C (p.Ser1436=)	rs1060915	Synonymous SNV	Benign	c.4308T>C (p.Ser1436=)	rs1060915	Synonymous SNV	Benign
				c.3548A>G (p.Lys1183Arg)	rs16942	Non-synonymous SNV	Benign	c.3548A>G (p.Lys1183Arg)	rs16942	Non-synonymous SNV	Benign
				c.3113A>G (p.Glu1038Gly)	rs16941	Non-synonymous SNV	Benign	c.3113A>G (p.Glu1038Gly)	rs16941	Non-synonymous SNV	Benign
				c.2612C>T (p.Pro871Leu)	rs799917	Non-synonymous SNV	Benign	c.2612C>T (p.Pro871Leu)	rs799917	Non-synonymous SNV	Benign
				c.2470_2471insTTCCGATCTTAGTCC (p.Pro 824delinsLPILVP)	-	Non-frameshift insertion	VUS	c.2470_2471insTTCCGATCTTAGTCC (p.Pro 824delinsLPILVP)	-	Non-frameshift insertion	VUS
				c.2311T>C (p.Leu771=)	rs16940	Synonymous SNV	Benign	c.2311T>C (p.Leu771=)	rs16940	Synonymous SNV	Benign
				c.2082C>T (p.Ser694=)	rs1799949	Synonymous SNV	Benign	c.2082C>T (p.Ser694=)	rs1799949	Synonymous SNV	Benign
S7	CP	88	PR	***BRCA2***							
				c.3396A>G (p.Lys1132=)	rs1801406	Synonymous SNV	Little Clinical Significance	c.3396A>G (p.Lys1132=)	rs1801406	Synonymous SNV	Little Clinical Significance
				c.4563A>G (p.Leu1521=)	rs206075	Synonymous SNV	Benign	c.4563A>G (p.Leu1521=)	rs206075	Synonymous SNV	Benign
				c.6513G>C (p.Val2171=)	rs206076	Synonymous SNV	Benign	c.6513G>C (p.Val2171=)	rs206076	Synonymous SNV	Benign
				c.7397T>C (p.Val2466Ala)	rs169547	Non-synonymous SNV	Benign	c.7397T>C (p.Val2466Ala)	rs169547	Non-synonymous SNV	Benign
				c.3823_3824insAGCAGTTCC (p.Ile1275delinsKQFL)	-	Frameshift insertion	VUS	c.3823_3824insAGCAGTTCC (p.Ile1275delinsKQFL)	-	Frameshift insertion	VUS
				c.3824_3825del p.(Ile1275ArgfsTer4)	-	Frameshift deletion	Pathogenic	c.3824_3825del p.(Ile1275ArgfsTer4)	-	Frameshift deletion	Pathogenic
				c.8878C>T p.(Gln2960Ter)	-	Non-synonymous SNV	Pathogenic	c.8878C>T p.(Gln2960Ter)	-	Non-synonymous SNV	Pathogenic

				c.8881_8884del (p.Gly2961fs)	-	Frameshift deletion	VUS	c.8881_8884del (p.Gly2961fs)	-	Frameshift deletion	VUS
				c.8885T>A	-	Stopgain	VUS	c.8885T>A	-	Stopgain	VUS
				No mutations				c.3807T>C (p.Val1269=)	rs543304	Synonymous SNV	Likely benign
				PALB2							
				No mutations				c.3114-51T>A	rs249936	Intronic	Benign
				No mutations				c.747T>A (p.Pro249=)	-	Synonymous SNV	VUS
S8	CP	68	PR	***BRCA1***							
				No mutations				No mutations			
				BRCA2							
				c.1114A>C (p.Asn372His)	rs144848	Non-synonymous SNV	Little Clinical Significance	c.1114A>C (p.Asn372His)	rs144848	Non-synonymous SNV	Little Clinical Significance
				c.3823_3824insAGCAGTTCC (p.Ile1275delinsKQFL)	-	Frameshift insertion	VUS	c.3823_3824insAGCAGTTCC (p.Ile1275delinsKQFL)	-	Frameshift insertion	VUS
				c.3824_3825del p.(Ile1275ArgfsTer4)	-	Frameshift deletion	Pathogenic	c.3824_3825del p.(Ile1275ArgfsTer4)	-	Frameshift deletion	Pathogenic
				c.4563A>G (p.Leu1521=)	rs206075	Synonymous SNV	Benign	c.4563A>G (p.Leu1521=)	rs206075	Synonymous SNV	Benign
				c.6513G>C (p.Val2171=)	rs206076	Synonymous SNV	Benign	c.6513G>C (p.Val2171=)	rs206076	Synonymous SNV	Benign
				c.7397T>C (p.Val2466Ala)	rs169547	Non-synonymous SNV	Benign	c.7397T>C (p.Val2466Ala)	rs169547	Non-synonymous SNV	Benign
				c.9090delA (p.T3030fs)	rs397507420	Frameshift deletion	Pathogenic	c.9090delA (p.T3030fs)	rs397507420	Frameshift deletion	Pathogenic
				c.9090dupA (p.Thr3030fs)	-	Frameshift duplication	VUS	c.9090dupA (p.Thr3030fs)	-	Frameshift duplication	VUS
				c.8878C>T p.(Gln2960Ter)	-	Non-synonymous SNV	Pathogenic	Elimination			
				c.8881_8884del (p.Gly2961fs)	-	Frameshift deletion	VUS				
				c.8885T>A	-	Stopgain	VUS				
				PALB2							
				c.3114-51T>A	rs249936	Intronic	Benign	c.3114-51T>A	rs249936	Intronic	Benign
				c.2586+58C>T	rs249954	Intronic	Benign	c.2586+58C>T	rs249954	Intronic	Benign
				c.1706_1707del (p.Lys569fs)	rs1060502759	Frameshift deletion	Pathogenic	Elimination			
				c.1706delA (p.Lys569fs)	-	Frameshift deletion	VUS	Elimination			
				No mutations				c.1676A>G (p.Gln559Arg)	rs152451	Non-synonymous SNV	Benign
				No mutations				c.1675_1676insGAGTGAAAGGTAAATCAAGATGTGTGCTCTTCCGACTCC (p.Q559delinsRVKGKSRCVLFRLQ)	-	Non-frameshift insertion	VUS
S9	CP	86	PR	***BRCA1***							
				No mutations				No mutations			
				BRCA2							
				c.1114A>C (p.Asn372His)	rs144848	Non-synonymous SNV	Little	c.1114A>C (p.Asn372His)	rs144848	Non-synonymous SNV	Little

							Clinical Significance				Clinical Significance
				c.4563A>G (p.Leu1521=)	rs206075	Synonymous SNV	Benign	c.4563A>G (p.Leu1521=)	rs206075	Synonymous SNV	Benign
				c.6513G>C (p.Val2171=)	rs206076	Synonymous SNV	Benign	c.6513G>C (p.Val2171=)	rs206076	Synonymous SNV	Benign
				c.7397T>C (p.Val2466Ala)	rs169547	Non-synonymous SNV	Benign	c.7397T>C (p.Val2466Ala)	rs169547	Non-synonymous SNV	Benign
				No mutations				c.3823_3824insAGCAGTTCC (p.Ile1275delinsKQFL)	-	Frameshift insertion	VUS
								c.3824_3825del p.(Ile1275ArgfsTer4)	-	Frameshift deletion	Pathogenic
				PALB2							
				c.3114-51T>A	rs249936	Intronic	Benign	c.3114-51T>A	rs249936	Intronic	Benign
V4	CP	100	CR	***BRCA1***							
				c.4900A>G (p.Ser1634Gly)	rs1799966	Non-synonymous SNV	Benign	c.4900A>G (p.Ser1634Gly)	rs1799966	Non-synonymous SNV	Benign
				c.4308T>C (p.Ser1436=)	rs1060915	Synonymous SNV	Benign	c.4308T>C (p.Ser1436=)	rs1060915	Synonymous SNV	Benign
				c.3548A>G (p.Lys1183Arg)	rs16942	Non-synonymous SNV	Benign	c.3548A>G (p.Lys1183Arg)	rs16942	Non-synonymous SNV	Benign
				c.3113A>G (p.Glu1038Gly)	rs16941	Non-synonymous SNV	Benign	c.3113A>G (p.Glu1038Gly)	rs16941	Non-synonymous SNV	Benign
				c.2612C>T (p.Pro871Leu)	rs799917	Non-synonymous SNV	Benign	c.2612C>T (p.Pro871Leu)	rs799917	Non-synonymous SNV	Benign
				c.2470_2471insTTCCGATCTTAGTCC (p.Pro 824delinsLPILVP)	-	Non-frameshift insertion	VUS	c.2470_2471insTTCCGATCTTAGTCC (p.Pro 824delinsLPILVP)	-	Non-frameshift insertion	VUS
				c.2609C>G (p.Ala870Gly)	rs1060502324	Non-synonymous SNV	VUS	Elimination			
				c.2608G>A (p.Ala870Thr)	rs753256448	Non-synonymous SNV	VUS				
				c.2311T>C (p.Leu771=)	rs16940	Synonymous SNV	Benign	c.2311T>C (p.Leu771=)	rs16940	Synonymous SNV	Benign
				c.2082C>T (p.Ser694=)	rs1799949	Synonymous SNV	Benign	c.2082C>T (p.Ser694=)	rs1799949	Synonymous SNV	Benign
				BRCA2							
				c.1114A>C (p.Asn372His)	rs144848	Non-synonymous SNV	Little Clinical Significance	c.1114A>C (p.Asn372His)	rs144848	Non-synonymous SNV	Little Clinical Significance
				c.4563A>G (p.Leu1521=)	rs206075	Synonymous SNV	Benign	c.4563A>G (p.Leu1521=)	rs206075	Synonymous SNV	Benign
				c.6513G>C (p.Val2171=)	rs206076	Synonymous SNV	Benign	c.6513G>C (p.Val2171=)	rs206076	Synonymous SNV	Benign
				c.7397T>C (p.Val2466Ala)	rs169547	Non-synonymous SNV	Benign	c.7397T>C (p.Val2466Ala)	rs169547	Non-synonymous SNV	Benign
				c.4288_4289insGGAACTGAGT (p.Thr1430_1431Ala_delinsRNX)	-	Stopgain	VUS	Elimination			
				No mutations				c.3823_3824insAGCAGTTCC (p.Ile1275delinsKQFL)	-	Frameshift insertion	VUS
								c.3824_3825del p.(Ile1275ArgfsTer4)	-	Frameshift deletion	Pathogenic
				PALB2							
				c.3114-51T>A	rs249936	Intronic	Benign	Elimination			
				c.2552delA (p.Asn851fs)	-	Frameshift deletion	VUS	Elimination			
				c.1288delC (p.Gln467fs)	-	Frameshift deletion	VUS	Elimination			

T1	CP	80	PR	***BRCA1***							
				c.4900A>G (p.Ser1634Gly)	rs1799966	Non-synonymous SNV	Benign	c.4900A>G (p.Ser1634Gly)	rs1799966	Non-synonymous SNV	Benign
				c.4308T>C (p.Ser1436=)	rs1060915	Synonymous SNV	Benign	c.4308T>C (p.Ser1436=)	rs1060915	Synonymous SNV	Benign
				c.3548A>G (p.Lys1183Arg)	rs16942	Non-synonymous SNV	Benign	c.3548A>G (p.Lys1183Arg)	rs16942	Non-synonymous SNV	Benign
				c.3113A>G (p.Glu1038Gly)	rs16941	Non-synonymous SNV	Benign	c.3113A>G (p.Glu1038Gly)	rs16941	Non-synonymous SNV	Benign
				c.2612C>T (p.Pro871Leu)	rs799917	Non-synonymous SNV	Benign	c.2612C>T (p.Pro871Leu)	rs799917	Non-synonymous SNV	Benign
				c.2470_2471insTTCCGATCTTAGTCC (p.Pro 824delinsLPILVP)	-	Non-frameshift insertion	VUS	c.2470_2471insTTCCGATCTTAGTCC (p.Pro 824delinsLPILVP)	-	Non-frameshift insertion	VUS
				c.2311T>C (p.Leu771=)	rs16940	Synonymous SNV	Benign	c.2311T>C (p.Leu771=)	rs16940	Synonymous SNV	Benign
				c.2082C>T (p.Ser694=)	rs1799949	Synonymous SNV	Benign	c.2082C>T (p.Ser694=)	rs1799949	Synonymous SNV	Benign
				No mutations				c.2609C>G (p.Ala870Gly)	rs1060502324	Non-synonymous SNV	VUS
								c.2608G>A (p.Ala870Thr)	rs753256448	Non-synonymous SNV	VUS
				BRCA2							
				c.1114A>C (p.Asn372His)	rs144848	Non-synonymous SNV	Little Clinical Significance	c.1114A>C (p.Asn372His)	rs144848	Non-synonymous SNV	Little Clinical Significance
				c.4563A>G (p.Leu1521=)	rs206075	Synonymous SNV	Benign	c.4563A>G (p.Leu1521=)	rs206075	Synonymous SNV	Benign
				c.6513G>C (p.Val2171=)	rs206076	Synonymous SNV	Benign	c.6513G>C (p.Val2171=)	rs206076	Synonymous SNV	Benign
				c.7397T>C (p.Val2466Ala)	rs169547	Non-synonymous SNV	Benign	c.7397T>C (p.Val2466Ala)	rs169547	Non-synonymous SNV	Benign
				c.4288_4289insGGAACTGAGT (p.Thr1430_1431Ala_delinsRNX)	-	Stopgain	VUS	c.4288_4289insGGAACTGAGT (p.Thr1430_1431Ala_delinsRNX)	-	Stopgain	VUS
				No mutations				c.8878C>T p.(Gln2960Ter)	-	Non-synonymous SNV	Pathogenic
								c.8881_8884del (p.Gly2961fs)	-	Frameshift deletion	VUS
								c.8885T>A	-	Stopgain	VUS
				c.7108_7109insCAT (p.K2370delinsTX)	-	Stopgain	VUS	Elimination			
				c.7110_7111insATATGTGGG (p.Lys2370delinsKICG)	-	Non-frameshift insertion	VUS				
				c.3807T>C (p.Val1269=)	rs543304	Synonymous SNV	Likely benign				
				c.9090delA (p.T3030fs)	rs397507420	Frameshift deletion	Pathogenic				
				c.9090dupA (p.Thr3030fs)	-	Frameshift duplication	VUS				
				PALB2							
				c.3351-53delT	rs35294437	Intronic	VUS	c.3351-53delT	rs35294437	Intronic	VUS
				No mutations				c.3114-51T>A	rs249936	Intronic	Benign
Ts1	CP	50	PR	***BRCA1***							
				c.4900A>G (p.Ser1634Gly)	rs1799966	Non-synonymous SNV	Benign	c.4900A>G (p.Ser1634Gly)	rs1799966	Non-synonymous SNV	Benign
				c.4308T>C (p.Ser1436=)	rs1060915	Synonymous SNV	Benign	c.4308T>C (p.Ser1436=)	rs1060915	Synonymous SNV	Benign
				c.3548A>G (p.Lys1183Arg)	rs16942	Non-synonymous SNV	Benign	c.3548A>G (p.Lys1183Arg)	rs16942	Non-synonymous SNV	Benign

				c.3113A>G (p.Glu1038Gly)	rs16941	Non-synonymous SNV	Benign	c.3113A>G (p.Glu1038Gly)	rs16941	Non-synonymous SNV	Benign
				c.2612C>T (p.Pro871Leu)	rs799917	Non-synonymous SNV	Benign	c.2612C>T (p.Pro871Leu)	rs799917	Non-synonymous SNV	Benign
				c.2470_2471insTTCCGATC TTAGTCC (p.Pro 824delinsLPILVP)	-	Non-frameshift insertion	VUS	c.2470_2471insTTCCGATC TTAGTCC (p.Pro 824delinsLPILVP)	-	Non-frameshift insertion	VUS
				c.2311T>C (p.Leu771=)	rs16940	Synonymous SNV	Benign	c.2311T>C (p.Leu771=)	rs16940	Synonymous SNV	Benign
				c.4807_4821del (p.Pro1603_Val1607del)	rs80359888	Non-frameshift deletion	VUS	Elimination			
				No mutations				c.2609C>G (p.Ala870Gly)	rs1060502324	Non-synonymous SNV	VUS
								c.2608G>A (p.Ala870Thr)	rs753256448	Non-synonymous SNV	VUS
								c.2082C>T (p.Ser694=)	rs1799949	Synonymous SNV	Benign
				BRCA2							
				c.3396A>G (p.Lys1132=)	rs1801406	Synonymous SNV	Little Clinical Significance	c.3396A>G (p.Lys1132=)	rs1801406	Synonymous SNV	Little Clinical Significance
				c.4288_4289insGGAACTGA GT (p.Thr1430_1431Ala_delinsR NX)	-	Stopgain	VUS	c.4288_4289insGGAACTG AGT (p.Thr1430_1431Ala_delins RNX)	-	Stopgain	VUS
				c.4563A>G (p.Leu1521=)	rs206075	Synonymous SNV	Benign	c.4563A>G (p.Leu1521=)	rs206075	Synonymous SNV	Benign
				c.6513G>C (p.Val2171=)	rs206076	Synonymous SNV	Benign	c.6513G>C (p.Val2171=)	rs206076	Synonymous SNV	Benign
				c.7108_7109insCAT (p.K2370delinsTX)	-	Stopgain	VUS	c.7108_7109insCAT (p.K2370delinsTX)	-	Stopgain	VUS
				c.7110_7111insATATGTGG G (p.Lys2370delinsKICG)	-	Non-frameshift insertion	VUS	c.7110_7111insATATGTGG G (p.Lys2370delinsKICG)	-	Non-frameshift insertion	VUS
				c.7397T>C (p.Val2466Ala)	rs169547	Non-synonymous SNV	Benign	c.7397T>C (p.Val2466Ala)	rs169547	Non-synonymous SNV	Benign
				c.8878C>T p.(Gln2960Ter)	-	Non-synonymous SNV	Pathogenic	Elimination			
				c.8881_8884del (p.Gly2961fs)	-	Frameshift deletion	VUS				
				c.8885T>A	-	Stopgain	VUS				
				c.9090delA (p.T3030fs)	rs397507420	Frameshift deletion	Pathogenic				
				c.9090dupA (p.Thr3030fs)	-	Frameshift duplication	VUS				
				No mutations				c.7242A>G (p.Ser2414=)	rs1799955	Synonymous SNV	Little Clinical Significance
				PALB2							
				c.3114-51T>A	rs249936	Intronic	Benign	c.3114-51T>A	rs249936	Intronic	Benign
				c.2552delA (p.Asn851fs)	-	Frameshift deletion	VUS	Elimination			
				No mutations				c.921delA (p. Lys307fs)	rs202151522	Frameshift deletion	VUS
G2	CP	100	CR	***BRCA1***							
				c.2612C>T (p.Pro871Leu)	rs799917	Non-synonymous SNV	Benign	No date			
				c.2470_2471insTTCCGATC	-	Non-frameshift	VUS				

				TTAGTCC (p.Pro 824delinsLPILVP)		insertion		
				BRCA2				
				c.1114A>C (p.Asn372His)	rs144848	Non-synonymous SNV	Little Clinical Significance	No date
				c.4288_4289insGGAACTGAGT (p.Thr1430_1431Ala_delinsRNX)	-	Stopgain	VUS	
				c.4563A>G (p.Leu1521=)	rs206075	Synonymous SNV	Benign	
				c.6513G>C (p.Val2171=)	rs206076	Synonymous SNV	Benign	
				c.7108_7109insCAT (p.K2370delinsTX)	-	Stopgain	VUS	
				c.7110_7111insATATGTGGG (p.Lys2370delinsKICG)	-	Non-frameshift insertion	VUS	
				c.7397T>C (p.Val2466Ala)	rs169547	Non-synonymous SNV	Benign	
				c.8878C>T p.(Gln2960Ter)	-	Non-synonymous SNV	Pathogenic	
				c.8881_8884del (p.Gly2961fs)	-	Frameshift deletion	VUS	
				c.8885T>A	-	Stopgain	VUS	
				PALB2				
				c.3114-51T>A	rs249936	Intronic	Benign	No date
				c.2586+58C>T	rs249954	Intronic	Benign	
K6	CP	70	PR	***BRCA1***				
				c.4900A>G (p.Ser1634Gly)	rs1799966	Non-synonymous SNV	Benign	No date
				c.4308T>C (p.Ser1436=)	rs1060915	Synonymous SNV	Benign	
				c.3548A>G (p.Lys1183Arg)	rs16942	Non-synonymous SNV	Benign	
				c.3113A>G (p.Glu1038Gly)	rs16941	Non-synonymous SNV	Benign	
				c.2612C>T (p.Pro871Leu)	rs799917	Non-synonymous SNV	Benign	
				c.2470_2471insTTCCGATCTTAGTCC (p.Pro 824delinsLPILVP)	-	Non-frameshift insertion	VUS	
				c.2311T>C (p.Leu771=)	rs16940	Synonymous SNV	Benign	
				c.2082C>T (p.Ser694=)	rs1799949	Synonymous SNV	Benign	
				BRCA2				
				c.1114A>C (p.Asn372His)	rs144848	Non-synonymous SNV	Little Clinical Significance	No date
				c.3396A>G (p.Lys1132=)	rs1801406	Synonymous SNV	Little Clinical Significance	

				c.3823_3824insAGCAGTTCC (p.Ile1275delinsKQFL)	-	Frameshift insertion	VUS	
				c.3824_3825del p.(Ile1275ArgfsTer4)	-	Frameshift deletion	Pathogenic	
				c.4563A>G (p.Leu1521=)	rs206075	Synonymous SNV	Benign	
				c.6513G>C (p.Val2171=)	rs206076	Synonymous SNV	Benign	
				c.7242A>G (p.Ser2414=)	rs1799955	Synonymous SNV	Little Clinical Significance	
				c.7397T>C (p.Val2466Ala)	rs169547	Non-synonymous SNV	Benign	
				c.8878C>T p.(Gln2960Ter)	-	Non-synonymous SNV	Pathogenic	
				c.8881_8884del (p.Gly2961fs)	-	Frameshift deletion	VUS	
				c.8885T>A	-	Stopgain	VUS	
				c.9090delA (p.T3030fs)	rs397507420	Frameshift deletion	Pathogenic	
				c.9090dupA (p.Thr3030fs)	-	Frameshift duplication	VUS	
				PALB2				
				c.3351-53delT	rs35294437	Intronic	VUS	No date

*Note: CR - complete regression; PR - partial regression, ST - stabilization; P - progression; # - did not perform sequencing of surgical material; * - rs397855890 was merged into rs35294437 on October 12, 2018 (Build 152); ** - rs780210723 was merged into rs35294437 on October 12, 2018 (Build 152); VUS - variants of uncertain significance*
